# Microbiological toxicity tests using standardized ISO/OECD methods—current state and outlook

**DOI:** 10.1007/s00253-024-13286-0

**Published:** 2024-08-31

**Authors:** Uwe Strotmann, Marie-José Durand, Gerald Thouand, Christian Eberlein, Hermann J. Heipieper, Stefan Gartiser, Udo Pagga

**Affiliations:** 1 Dept. of Chemistry, Westfälische Hochschule, Recklinghausen, Germany; 2 UMR 6144, Nantes Université, ONIRIS, CNRS, GEPEA, 85000 La Roche Sur Yon, France; 3 Department of Molecular Environmental Biotechnology, Helmholtz Centre for Environmental Research - UFZ, Leipzig, Germany; 4 Hydrotox GmbH, Bötzinger Str. 29, 79111 Freiburg, Germany; 5 Rüdigerstr. 49, 67069 Ludwigshafen, Germany

**Keywords:** Microbial toxicity, Biodegradation, Standardized ISO and OECD tests, Biodegradation adaptation potential (BAP), Chemical resistance potential (CRP), Physiological potential of an inoculum (PPI), Wastewater treatment plant, Toxicity monitoring, Toximeter

## Abstract

**Abstract:**

Microbial toxicity tests play an important role in various scientific and technical fields including the risk assessment of chemical compounds in the environment. There is a large battery of normalized tests available that have been standardized by ISO (International Organization for Standardization) and OECD (Organization for Economic Co-operation and Development) and which are worldwide accepted and applied. The focus of this review is to provide information on microbial toxicity tests, which are used to elucidate effects in other laboratory tests such as biodegradation tests, and for the prediction of effects in natural and technical aqueous compartments in the environment. The various standardized tests as well as not normalized methods are described and their advantages and disadvantages are discussed. In addition, the sensitivity and usefulness of such tests including a short comparison with other ecotoxicological tests is presented. Moreover, the far-reaching influence of microbial toxicity tests on biodegradation tests is also demonstrated. A new concept of the physiological potential of an inoculum (PPI) consisting of microbial toxicity tests whose results are expressed as a chemical resistance potential (CRP) and the biodegradation adaptation potential (BAP) of an inoculum is described that may be helpful to characterize inocula used for biodegradation tests.

**Key points:**

• *Microbial toxicity tests standardized by ISO and OECD have large differences in sensitivity and applicability.*

• *Standardized microbial toxicity tests in combination with biodegradability tests open a new way to characterize inocula for biodegradation tests.*

• *Standardized microbial toxicity tests together with ecotoxicity tests can form a very effective toolbox for the characterization of toxic effects of chemicals.*

**Supplementary Information:**

The online version contains supplementary material available at 10.1007/s00253-024-13286-0.

## Introduction

**Fig. 1:**
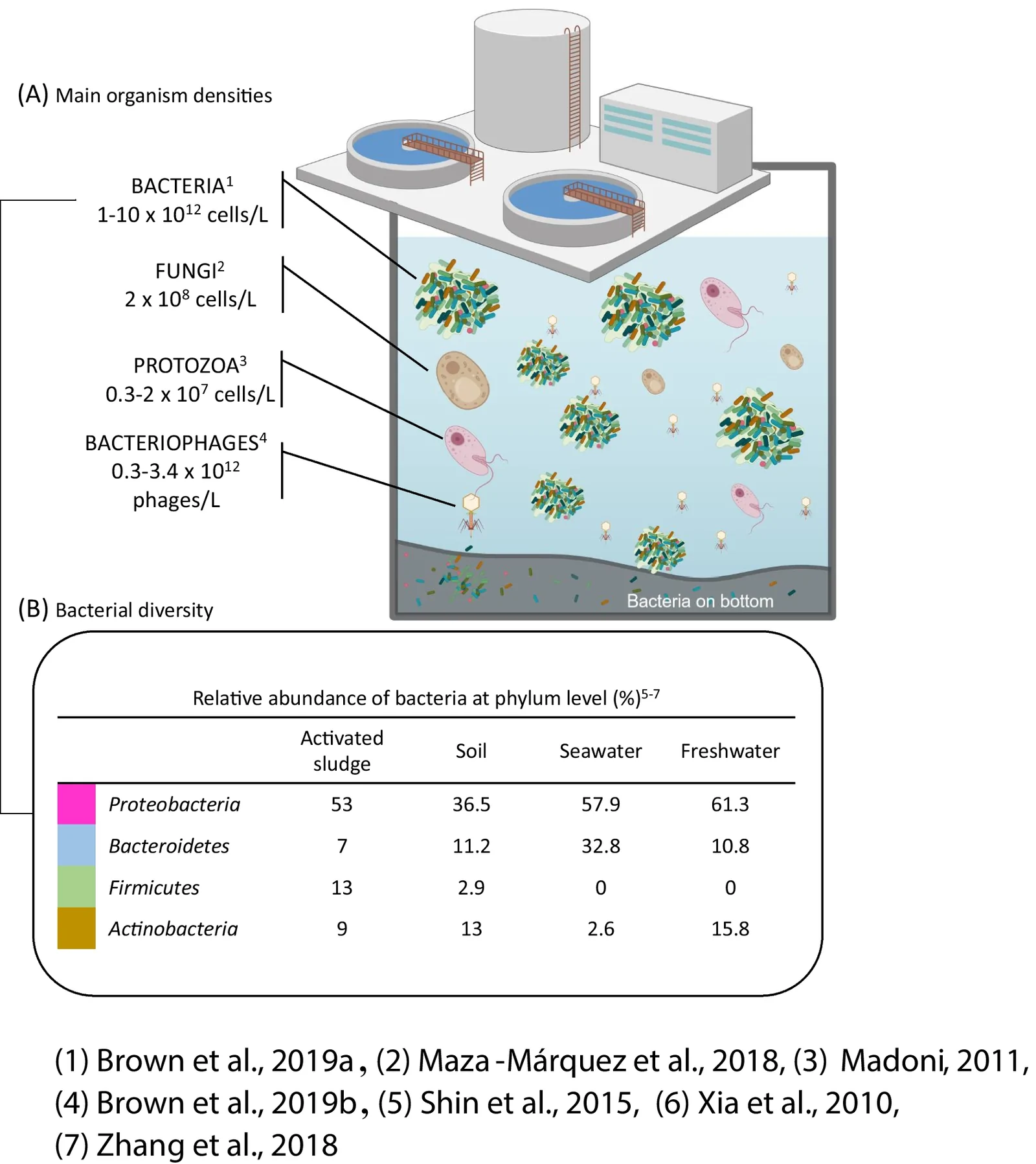
Main organism groups present in activated sludge. Also, the main bacterial *phyla* (*Proteobacteria, Bacteroides, Firmicutes, and Actinobacteria*) present in different environmental compartments are indicated. References: 1 (Brown et al. 2019a), 2 (Maza-Marquez et al. 2018), 3 (Madoni 2011), 4 (Brown et al. 2019b), 5 (Shin et al. 2015), 6 (Xia et al. 2010), 7 (Zhang et al. 2018)

**Fig. 2:**
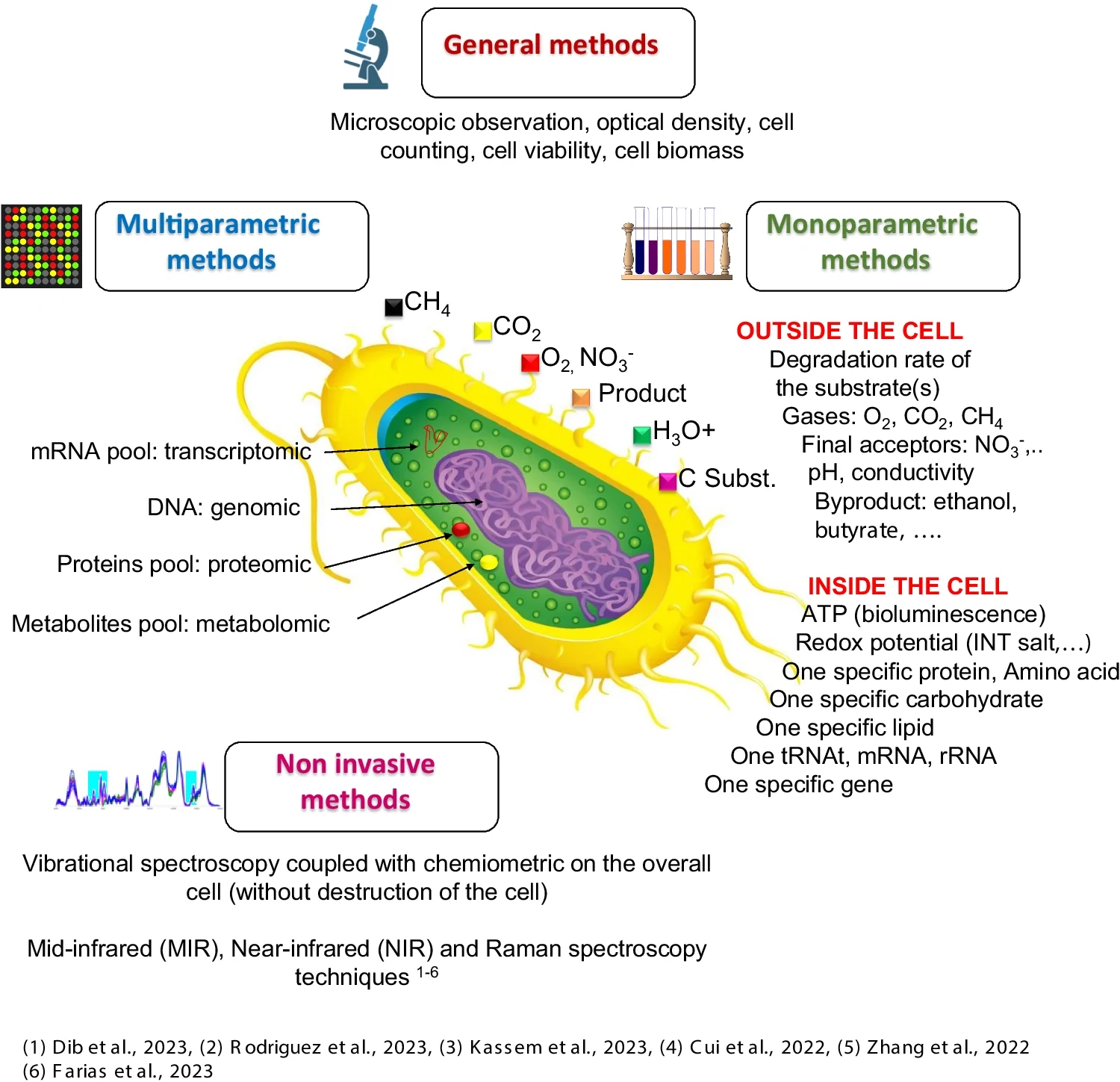
Overall description of the methods used to characterize bacterial cells (the graphics of the bacteria cell was retooled from Vectorstock) References: 1 (Dib et al. 2023), 2 (Rodriguez et al. 2023), 3 (Kassem et al. 2023), 4 (Cui et al. 2022), 5 (Zhang et al. 2022), 6 (Farias et al. 2023)

An increasing number of chemical compounds are being produced worldwide in large amounts (CEFIC 2023; Scheringer et al. 2012; Strempel et al. 2012; Wang et al. 2020). It is very important to know their fate and ecotoxicological behavior in the environment in order to make predictions and to take appropriate measures to prevent harmful effects. Biodegradability as well as possible toxic effects and bioaccumulation are the most important criteria for this purpose. In different recent reviews, this matter has been stressed and available test methods have been discussed (Kowalczyk et al. 2015; Strotmann et al. 2023). An effective assessment of chemical compounds for the aquatic compartment, e.g., under REACH (registration, evaluation, authorization, and restriction of chemicals) is mainly based of acute and chronic tests with representative organisms from different trophic levels (algae, crustacea, fish). Toxicity against bacteria is only routinely assessed for the compartment of wastewater treatment plants (WWTPs), often applying the activated sludge respiration inhibition test according to (ISO 8192 2007; OECD 209 2010) and the nitrification inhibition test (ISO 9509 2006; OECD 209 2010). However, bacterial toxicity is also an important factor when interpreting data from biodegradability tests and analyzing biotechnological processes. Activated sludge from WWTPs is the inoculum most often used in tests for determining biodegradability. The species composition of the activated sludge is very diverse and difficult to determine. An overview of the organisms present (Fig. 1) shows that bacteria play a predominant role. The possibilities to identify and characterize bacterial species are limited and vary from classical microbiological methods, over phenotypical, and molecular biological methods up to new spectroscopic techniques (Fig. 2). The importance of bacteria in this complex system cannot be overestimated and inhibitory effects can have far-reaching consequences. Therefore, many test methods for determining potential bacterial toxicity exist which use different inocula, species, and endpoints.

The importance of biodegradability has been pointed out in an overview where also current test methods have been listed (Strotmann et al. 2023). Microbial toxicity tests can also serve for analyzing biotechnological processes in wastewater treatment such as nitrification, denitrification, and nitrogen elimination as well as the control of toxicity of influent and effluent in WWTPs.

In order not to hinder or prevent degradation processes in the natural environment and in technical facilities, some important criteria must be met. One is the toxic effect of chemicals or wastewaters on microorganisms, especially on bacteria, because they are primarily responsible for biological degradation and, therefore, also for their elimination in the environment. In this context, the determination of possible toxic effects of wastewaters and UVCBs (substances of unknown or variable composition, complex reaction products, or biological materials) using appropriate test methods and the correct evaluation of the test results is an important task. Especially UVCBs have come into focus in the last years. They include chemical mixtures such as detergents, fragrances, and personal care ingredients. But they also serve as fuel and are used for chemical reactions. It also has to be mentioned that about 20 to 40% of the chemicals registered in Europe and the USA are UVCBs (Lai et al. 2022). Therefore, this class of chemical mixtures poses large future challenges concerning persistence assessment including the estimation of biodegradability and microbial toxicity (Dimitrov et al. 2015; Kutsarova et al. 2019; Prosser et al. 2023).

Up to now, a number of biotests have been developed to detect possible toxic effects of chemicals in aqueous systems and to make predictions for natural environments (For reviews, see Escher et al. 2021). As a practical consequence, there are some methods available to measure the quality of effluents of WWTPs. They cover a wide range of possible effects such as cytotoxicity, genotoxicity, estrogenicity, and androgenicity (Bain et al. 2014; Bertanza et al. 2013, 2022; Carvalho et al. 2022; Escher et al. 2014; Leusch et al. 2010, 2014; Smital et al. 2011; Stalter et al. 2011; Välitalo et al. 2017). There also exist methods for determining bacterial toxicity using various defined species and endpoints, but their regulatory significance is limited and their use is a particular challenge. It is not the technical side, which poses the problems. There are simple, reliable laboratory methods available, and even dynamic test systems are known to continuously monitor complex systems such as wastewaters. It is rather the selection of the right organisms and the evaluation of the test results, which plays a crucial role. With microorganisms, more than with other organisms, the question arises, which bacterial species or which mixture of microorganisms, the so-called inoculum (for example, activated sludge in biological WWTPs), should be used to represent the huge abundance of bacteria in the natural and technical environment and their intrinsic enormous biochemical and physiological potential (Wu et al. 2019).

Therefore, the targets of microbial toxicity testing are also very diverse (Bitton and Dutka 1986; Dutka and Bitton 1986; Pagga and Strotmann 1999). Toxic compounds can have an influence on enzymes in the catabolic, anabolic, and intermediate metabolism, on nucleic acids, on membranes and the cell wall as well as on the floc structure in activated sludge. In some cases, organic compounds are toxic for living organisms because they accumulate in cell membranes and damage these. Furthermore, essential metabolic processes such as respiration and cell growth, nitrification, or denitrification can also be inhibited, which can be checked and prevented by appropriate test methods. Random processes such as the inhibition of light emission by bioluminescent bacteria (e.g., *Aliivibrio fischeri)* are used to determine toxic effects on microorganisms. One should take note that the use of pure bacterial cultures (e.g., *Pseudomonas* species), was one of the earliest approaches in the field of bacterial toxicity, but these methods are only of limited value, because in technical facilities, such as WWTPs, the main goal of tests and predictions are focused on activated sludge which is always a complex microbial culture.

This article provides an overview of available standardized and non-standardized microbial toxicity tests with bacteria, the evaluation of the test results, and a short discussion of future possibilities. However, these tests do not only affect natural and technical environmental systems as they are also used as pre-tests for biodegradation tests or as parallel test assays. Furthermore, they may also be used for the characterization of the quality of the inocula used in these tests. This is especially true for ready biodegradability tests (RBTs) because these test results play a crucial role for the estimation of the environmental persistence of chemicals as well as for regulatory purposes (Gartiser et al. 2022; Kowalczyk et al. 2015; Pagga 1997; Painter 1995; Poursat et al. 2019; Strotmann et al. 2023). Microbial toxicity data of chemicals can also be compared with data from classical ecotoxicological tests, using for example fish, daphnia, or algae tests to enable a sound risk assessment in the environment. Furthermore, microbial toxicity tests play an important role in monitoring the influent and effluent quality of biological WWTPs the latter being a direct consequence of the biodegradation activity in the plant. Therefore, also intoxications in the event of technical disruptions of a treatment plant and unsuitable wastewater streams can be effectively monitored (Araujo et al. 2005; Bertanza et al. 2022; Oliveira et al. 2007; Pagga 1983).

## Importance and limitations of microbial toxicity tests

The metabolism of bacteria is extremely diverse, including both the use of various energy sources and different final degradation products. The determination of biodegradability is a fundamental task in the assessment of the environmental behavior of anthropogenically produced compounds. If the bacterial metabolism is severely inhibited by toxic substances, biodegradation processes may be impaired. This may not only lead to an enrichment of non-natural substances but may also have severe negative effects in natural environments such as surface waters, sediments, the marine environment, and soils. Moreover, also biotechnological processes which operate with degrading bacteria such as biological sewage treatment plants, anaerobic digesters, or composting sites may be negatively affected by toxic substances which can have far leading unfavorable consequences for the whole environment.

Laboratory toxicity tests with microorganisms are performed to predict a possible inhibition of biodegradation processes in environmental compartments. This helps for example to avoid disruptions in the operation of WWTPs or the deterioration of the self-cleaning ability of surface water, which may occur by the discharge of toxic wastewater or other toxic material. Useful results are obtained if essential aspects of the target environments are considered. Therefore, numerous factors have to be considered in the tests including the composition of the nutrient solution, salt concentration, pH value, temperature, and the oxygen supply. Furthermore, also the test duration, the concentration of the test substance, and the inoculum play a crucial role. Another important aspect is the biodegradation adaptation potential (BAP) of the inoculum, which is often determined by its origin (Strotmann et al. 2023). In order to avoid incorrect assessments of the test results it is advisable to determine possible inhibitory effects in advance or in parallel with biodegradation tests. Some degradation tests require a control assay using the test substance in combination with a well-known easily biodegradable substrate whose degradation should not be affected. Because of the great importance of the inoculum, its origin should be as far as possible comparable to the target compartment. For general statements, activated sludge used in toxicity or biodegradation tests should originate from standard municipal sewage treatment plants, but for monitoring a special plant treating for example chemical wastewater, it should be derived by that very plant. The reason is the better adaptation of the microorganisms in such a treatment plant which may be caused by a higher tolerance to toxic substances together with a higher biodegradation potential. In this ideal case, reliable predictions for the system to be monitored are possible. However, the transfer to other systems, for example, to other treatment plants or natural environments may cause problems. If even the transmission of test data with activated sludge is problematic, the use of pure bacterial cultures can be even more. Test systems with *Pseudomonas* species for example may be used to determine an intrinsic toxic potential of substances. On the other hand, it has to be considered, that bacteria show a wide metabolic variety depending on the habitat in which they naturally occur. A transfer of these data from a single species test to make predictions for a certain environmental compartment is rather difficult and may cause problems. For this reason, it is not possible to recommend one single microbial inhibition test that fits for all applications. However, a reasonable generalization can be made by using standardized test methods.

Another important point concerns microbial toxicity tests as pre-tests for biodegradation tests. It is advisable to perform such tests before biodegradation tests are carried out in order to know at which concentrations inhibiting effects may occur. The chosen test concentration should not exceed the EC_20_ value of the toxicity test, which is the concentration that causes an inhibition of 20 percent compared to the unaffected control. This information may also be obtained in parallel test vessels during the degradation tests (inhibitory control assays), where an easily biodegradable substance and the test substance are mixed. If degradation of the easily degradable substance is detected but not for the test substance, one can rule out that toxic effects may play a role. Furthermore, it can be checked if abiotic degradation or elimination from water occurs by adding a well-known, very toxic substance to one of the standardized test mixtures. A loss of the test substance can then only be due to abiotic processes such as evaporation or adsorption. For this purpose, mercury (II) chloride is often used as an inhibitor because of its strong and reproducible toxic effect. On the other hand, its practical usage in laboratories is critically viewed for reasons of a possible environmental pollution by discharging the residues of the tests.

## Overview of microbial toxicity tests

### Standardized bacterial toxicity tests (OECD/*ISO*)

Several standardized laboratory methods are available for testing bacterial toxicity, using both mixed cultures such as activated sludge and pure cultures such as *Pseudomonas putida* (for an overview, see Table 1). These are usually static test systems mainly representing a freshwater environment and which are operated under largely defined conditions including the test volume, the nutrient solution, the way of mixing, or in aerobic tests, the oxygen supply. The test duration ranges from a few minutes to a few hours. For pure cultures, a distinction must also be made between the duration of cultivation, the incubation with the test substance, and the actual measurement duration. Measured variables are usually the inhibition of respiration by the determination of oxygen consumption and the inhibition of growth via turbidity measurements. The main purpose is to predict the inhibition of bacterial growth as well as the inhibition of biodegradation processes. When the focus is on other important bacterial activities in the environment such as the inhibition of nitrification and denitrification, special test methods are required. In addition, anaerobic inhibition tests are applied, where the underlying principle is the inhibition of biogas production by chemical compounds, determined in comparison to a control without a test compound. A test method derived from the marine environment is the luminescent bacteria test, in which the inhibition of the light emission of special bacteria is used as an indication of toxic effects. The disadvantage of this test is that luminescent bacteria are organisms derived from the marine environment, which tolerate high salt concentrations. These bacteria are different to those from limnic systems such as rivers, lakes, or another surface water. It also has to be mentioned that the phenomenon of luminescence is closely coupled to the catabolic energy-producing metabolism, but that the light emission is not a prerequisite for the survival of the bacteria. The great advantage of the method is, however, that it can be performed rapidly without much effort and that it can be easily automatized. Tests with luminescent bacteria and pure bacterial cultures can at least give a reliable indication of the toxic potential of substances and its relative classification. In addition to these short-term tests, it may sometimes be necessary to carry out long-term studies of toxic environmental effects. On the other hand, these tests are much more complex and more time-consuming than simple laboratory tests and need very special test designs.

**Table 1:** Different OECD/ISO-based microbial toxicity tests

Test	Method	Test principle
Inhibition of heterotrophic respiration	(ISO 8192 2007; OECD 209 2010)	measurement of oxygen consumption due to heterotrophic respiration
Inhibition of nitrification inhibition	(ISO 9509 2006; OECD 209 2010)	measurement of oxygen consumption due to nitrification respiration (OECD 209) or ammonium oxidation, nitrite, and nitrate formation (ISO 9509)
Luminescent bacteria test	(ISO 11348 2008, ISO 21338 2010)	measurement of inhibition of light emission (ISO 11348) kinetic version of the luminescent bacteria test (for sediments, solids, and colored samples) (ISO 21338)
*Pseudomonas putida* growth inhibition test	(ISO 10712 1995)	measurement of growth inhibition of a growing pure culture of *Pseudomonas putida*
Growth inhibition test with activated sludge bacteria	(ISO 15522 1999)	measurement of growth inhibition of activated sludge bacteria
Determination of the inhibition of the activity of anaerobic bacteria	(ISO 13461 2003; OECD 224 2007)	measurement of inhibition of biogas production
Dehydrogenase activity	(ISO 18187 2023)	Measurement of dehydrogenase activity in a contact test with *Arthrobacter globiformis*
Nitrogen transformation in soil by microorganisms	(OECD 216 2000)	Measurement of the transformation of nitrogen in soils by microorganisms
Carbon transformation in soil by microorganisms	(OECD 217 2000)	Measurement of the transformation of carbon in soils by microorganisms
Transformation of organic matter in contaminated soils	(ISO 23265 2023)	Measurement of the transformation of organic matter in contaminated soils by microorganisms

The results of toxicity tests are usually expressed as limit values that are recorded as effective concentrations (EC_x_, with *x* representing the degree of inhibition in percent) in mM (mg/L). The beginning of a significant inhibition is for example an inhibition of 20% compared to a control without a test substance. In order to compare the inhibitory effects of different substances, the statistically more reliable EC_50_ value is the most important value. In order to indicate the concentration that causes a complete inhibition the EC_80_ value is generally used.

## Respiration inhibition tests

This type of bacterial inhibition test is based on the fact that the oxidative degradation of organic compounds is strictly coupled to a utilization of oxygen which acts as a terminal electron acceptor in the respiration chain. The following Eq. (1) gives a general overview of the overall reaction:

1 $$Carbon\;source+O_2+X\Rightarrow H_2O+CO_2+byproducts$$

The heterotrophic respiration (HR) is a vital process for energy generation and biomass production under aerobic conditions. Inhibition of this process by toxic compounds has severe consequences on the biodegradation processes of heterotrophic bacteria. Therefore, these types of inhibition tests are widely used before performing biodegradation tests and to monitor wastewater treatment plants. The standardized methods ISO 8192 and OECD 209 are also known as “short-term respiration inhibition test.” The preferred incubation time of ISO 8192 (e.g., used for wastewater samples) is 30 min while that of OECD 209 (e.g., used for chemical substances) is 3 h. But ISO 8192 also states that the incubation time may extended up to 180 min in the case of poorly water-soluble test materials and in special cases even up to 27 h (Gendig et al. 2003). As an inoculum activated sludge is used at a concentration of 1.5 g L^−1^. For general predictions, it is usually taken from municipal WWTPs. The inoculum may be pre-aerated to degrade excess carbon material. The oxygen concentration is measured with the help of oxygen electrodes. The test results are expressed as EC_20_, EC_50_, and EC_80_ or as a limit value which shows no inhibition. As a reference compound 3,5-dichlorophenol is frequently used. When the total respiration (TR; the sum of heterotrophic respiration and nitrification respiration) is determined the EC_50_ is at 9.8 mg L^−1^ (validity range: 2 to 25 mg L^−1^; ISO 8192 2007). Concerning the heterotrophic respiration, the EC_50_ is at 20.3 mg L^−1^ (validity range: 5 to 40 mg L^−1^ (ISO 8192 2007). As the test is very reliable and shows a good reproducibility it can be regarded as a standard microbial toxicity test for the estimation of a possible toxicity of chemicals. On the other hand, the sensitivity of the test can be increased when using a prolonged incubation time of 27 h (see Table S1). Especially for 2,3-dichlorophenol an increased sensitivity could be stated when using a test over 27 h with municipal sludge. The source of activated sludge can also have a significant influence (see Table S1). In case of uncertainty, it is therefore useful to perform tests with different incubation times and inocula from different sources. This test system is also very reliable when testing the toxicity of wastewaters (Oliveira et al. 2007; Pagga 1981).

## Nitrification inhibition test

The process of nitrification is an important natural process that occurs in soil and water. It involves the conversion of ammonia (NH_3_) or ammonium (NH_4_^+^) into nitrite (NO_2_^−^), and then into nitrate (NO_3_^−^) by autotrophic bacteria using molecular oxygen as a terminal electron acceptor in order to generate energy for their metabolism. As nitrifying bacteria have very low growth rate constants they can be easily washed out in wastewater treatment plants when they are not retained by adequate means (Tchobanoglous et al. 1991). The inhibition of nitrification can also be considered a suitable indication for any other toxic effects in microbial environments. Principally the test, as normalized by ISO, can be performed in two different ways. Firstly, the nitrification activity and inhibition of nitrification can be determined by an analysis of the decrease of ammonium and the subsequent production of nitrite and nitrate (ISO 9509 2006). Alternatively, the oxygen consumption due to this oxidation process can be determined (ISO 8192 2007). The nitrification respiration can be calculated by the following Eq. (2):

2 NR=TR-HR

where NR is the nitrification respiration, HR is the heterotrophic respiration and TR is the total respiration. Both the nitrification and the heterotrophic degradation of carbon compounds require oxygen. To distinguish between these processes and to selectively detect nitrification inhibition, allylthiourea (ATU) is used, because this substance selectively inhibits nitrification processes. In this way, the estimation of total respiration in an assay without ATU and the estimation of heterotrophic respiration in an assay containing ATU enables the determination of the nitrification respiration (NR). In addition, the results obtained can be verified by an analysis of nitrate formed by the oxidation of ammonium to nitrate via nitrite. In some cases, the bacterial oxidation of ammonium is not complete, but is stopped at the nitrite stage. The microbial background is that the whole nitrification process consists of two single processes, the nitritation (formation of nitrite from ammonia) and the nitration (subsequent formation of nitrate from nitrite). These processes are performed by the two groups of ammonia-oxidizing bacteria and nitrite-oxidizing bacteria. The two reactions of the whole nitrification process can be described by the following Eqs. 3 and 4:

3 $$2NH_4^++3O_2\Rightarrow2NO_2^-+4H^++2H_2O\rightarrow Formation\;of\;nitrite$$

4 $$2NO_2^-+O_2\Rightarrow2NO_3^-\rightarrow Formation\;of\;nitrate$$

The overall reaction can be described by the following Eq. (5):

5 $$2NH_4^++4O_2\Rightarrow2NO_3^-+4H^++2H_2O$$

From Eq. (5), it is obvious that there is a stochiometric proton release which requires sufficient buffering conditions in the test medium. Since this biological process is also very sensitive, nitrification inhibition tests are required to monitor the buffering capacity of the test medium.

In the ISO 9509 test system, the biomass concentration is at 1.5 g L^−1^, and the incubation time at least 4 h. The oxygen concentration has to be maintained at 4 mg L^−1^. Sodium hydrogencarbonate (NaHCO_3_) serves as an inorganic carbon source for the autotrophic nitrifiers. The pH value should be at 7.6. The inoculum is activated sludge from a suitable WWTP or from a special nitrifying enrichment culture. Nitrification activity is determined by chemical analyses of ammonium, nitrite, and nitrate. Reference compounds can be 3,5-dichlorophenol (EC_50_ = 5.6 mg L^−1^), 4-nitrophenol (EC_50_ = 43.3 mg L^−1^), or ATU (EC_50_ = 0.38 mg L^−1^) (ISO 9509 2006). It has to be mentioned that the EC_50_ values of a sludge from a wastewater treatment plant can be higher than from a nitrifying enrichment culture. Possible reasons are the adsorption processes of the inhibitors to sludge flocs and diffusion processes (ISO 9509 2006).

In the ISO 8192 test, the inhibition of nitrification can be determined by the measurement of the oxygen demand for nitrification processes according to Eq. 2. The EC_50_ value for 3,5-dichlorophenol determined in a ring test was at 4.6 mg L^−1^ (validity range: 0.1–10 mg L^−1^) which is quite similar to the values in the ISO 9509 test system.

Nitrification activity can also be measured using pure bacterial cultures, such as *Nitrosomas europaea* (NBRC 1429, ATCC 19718). Mizukami-Murata et al. (2023) and Nishigaya et al. (2016) studied the nitrification processes and reported a strong correlation with ISO 9509 and ISO 8192 for different chlorophenols and dichlorophenols showing a clear comparability of the methods. Comparative data published by Pagga et al. (2006) were in the same range, whereas Yuan et al. (2019) reported somewhat lower values in the range of 0.60 mg L^−1^.

## Growth inhibition tests

The growth inhibition test with activated sludge bacteria according to ISO 15522 specifies a method for assessing the potential toxicity of a test material to the growth of mixed aerobic bacteria present in activated sludge. The inhibitory effect is restricted to those microorganisms capable to grow with the chosen organic test medium. The standard outlines the test environment, reagents, apparatus, and the procedure to be followed. The underlying principle of the test is the exposition of mixed bacteria which originate from activated sludge to different concentrations of a test compound. The addition of the test compound to a nutrient mixture occurs in the early exponential phase. Growth is followed by turbidity measurements with a light of wavelength of 530 nm. The test was validated by testing phenol, different chlorophenols, and 2,4-dinitrophenol (Strotmann et al. 1994). In a subsequent ring test, the EC_50_ for 3,5-dichlorophenol proved to be at 8.1 mg L^−1^ and for KCN at 12.3 mg L^−1^ (Strotmann and Pagga 1996). Comparable data for 3,5-dichlorophenol were reported by Yuan et al. (2019) (EC_50_ = 4.22 mg L^−1^). The results of this test have a certain limitation, because the bacteria are cultivated on a complex medium containing nutrient broth and an easily degradable carbon source (e.g., sodium acetate) which is quite different from the composition of a real wastewater. Nevertheless, the EC_50_ values obtained are comparable to those of respiration inhibition tests.

The growth inhibition test with *Pseudomonas putida* (ISO 10712 1995) specifies a method similar to ISO 15522 for assessing the toxicity of wastewater and water-soluble substances to bacteria, but it uses pure cultures of a representative of heterotrophic microorganisms present in freshwaters. The toxic concentration range of the test substance is first determined in preliminary tests. In the main test, a serial dilution within the relevant concentration range is used with three replicates per concentration and two controls, a negative one without the test substance and a positive one with 3,5-dichlorophenol, which both are run in parallel. A defined bacterial suspension is added to each test vessel and after 16 ± 1 h, the cell concentration is determined by a turbidity measurement at a wavelength of 436 nm. Using this test the EC_50_ for 3,5-dichlorophenol was at 21.4 mg L^−1^ and slightly higher than in the ISO 15522 test with mixed bacteria. In former times, the *Pseudomonas putida* growth inhibition test was often used in Germany for the derivation of water-hazardous classes (WGK) of chemical substances. However, the system is no longer used and, additionally, the *Pseudomonas putida* strain has been classified into a risk group, which makes handling in laboratories considerably more difficult. It should be noted, that the OECD 201 2006 norm describes a growth inhibition test with algae and cyanobacteria within an incubation period of 72 h under defined lightening conditions. In fact, cyanobacteria (blue-green algae) such as *Anabaena flos-aquae* or *Synechococcus leopoliensis* are bacteria, but the test results are interpreted as algae toxicity data.

### Luminescent bacteria test

Light-emitting luminescent bacteria are very common in the marine environment. Their light emission is closely coupled to their catabolic metabolism and when it is disturbed by toxic compounds also the light emission decreases. This effect forms the basis of this inhibition test. Suitable bacterial strains are *Aliivibrio fischeri* (also designated as *Photobacterium phosphoreum* and *Vibrio phosphoreum)* and *Vibrio qinghaiensis* (Zhang et al. 2023)*.* The test system has been standardized in three variations (ISO 11348 Part 1 to 3). The different parts are due to the cultivation method of the bacterial strain (freshly cultivated, frozen, or lyophilized bacteria). When stored at a temperature of − 80 °C the bacteria keep their viability for years and can successfully be reactivated for the test (Strotmann et al. 2020). The performance of the luminescent bacteria test is easy and rapid and can simply be automated and even kits for simple use are commercially available. The luminescent bacteria test can be used to determine toxic effects of chemical compounds ranging from basic and intermediate chemicals to antibiotics and pesticides including fungicides, herbicides, and insecticides. Another important application is the supervision of all kinds of aqueous solutions ranging from drinking water, river, and surface water eluates up to process effluents and landfill leachates. It can also be used to determine the reduction of toxicity in wastewater treatment plants by analyzing the influent and effluent (Abbas et al. 2018; Araujo et al. 2005; Yuan et al. 2019). In some regard, the luminescent bacteria test is an ideal tool due to its short incubation time (5, 30, 60 min). Recently a modification of the original ISO method has been published where especially the cultivation and reactivation medium have been modified so that the performance of the test system is improved (Strotmann et al. 2020). Moreover, in this test, the commonly known reference compound 3,5-dichlorophenol is used. The EC_50_ values for different incubation times (5, 30, 60 min) are in a small range from 3.52 to 5.00 mg L^−1^ (Strotmann et al. 1994, 2020). In the ISO 11348 text, the results of a ring test are given where the EC_50_ is at 5.80 mg L^−1^. In a comparative study with different chlorophenols, dichlorophenols, and 2,4-dinitrophenol, it was found that 3,4 dichlorophenol was the most toxic (EC_50_ = 1 mg L^−1^) whereas phenol and 2.4-dinitrophenol exhibited the lowest toxicity (EC_50_ = 29 mg L^−1^ each) (Strotmann and Eglsäer 1995). The luminescent bacteria test is extremely popular and it is used in a large variety of applications ranging from toxicity determination of chemical compounds and mixtures of these up to a wide range of monitoring purposes (Abbas et al. 2018; Mendonca et al. 2009; Menz et al. 2013). Because of its simplicity and versatile application possibilities the luminescent bacteria test is a reliable screening test, which should be combined in certain situations of ambiguity with another microbial toxicity test which is not based on marine bacteria.

There also exists a kinetic version of the luminescent bacteria test (ISO 21338 2010; Lappalainen et al. 1999) which aims at the determination of light emission in sediments and other solids as well as in colored samples. Here, a kinetic measurement of light emission with its peak and its decay is recorded. The relevant parameter is the signal after a certain incubation time versus the height of the peak. Standard incubation times are 5, 15, and 30 min including a peak measurement 5 s after mixing the probe. Reference compounds are 3,5-dichlorophenol (EC_50_ = 5.1 mg L^−1^), zinc (II) (EC_50_ = 3.03 mg L^−1^), and chromium (VI) (EC_50_ = 15.3 mg L^−1^, 30 min) (Lappalainen et al. 2001).

## Inhibition controls in biodegradability tests

When there is a suspicion that a chemical substance or wastewater might have inhibitory effects to the inoculum in ready biodegradability tests, OECD 301 1992 recommends to consider additionally inhibitory control vessels, to avoid false negative conclusions. In the inhibition control, the test compound is incubated with a biodegradable reference substance. A reduction of the biodegradation of the reference substance is interpreted as a toxic effect to the inoculum used. If the biodegradation of the reference substance is less than 35% (based on total DOC) or less than 25% based on total theoretical oxygen demand (ThOD) or theoretical carbon dioxide evolution (ThCO_2_) within 14 days, the test substance is assumed to be inhibitory and the test should be repeated at a lower test concentration (OECD 301, Sect. 25). According to ECHA (European Chemicals Agency) Guidance for assessing potential toxic effects on microorganisms of activated sludge in WWTPs, the concentration tested in the inhibition control may be used for deriving the “no effect concentration” (NOEC) assuming an assessment factor (safety factor) of 10 (ECHA 2023). The fixed limit values are only a rough estimate. Often, a comparison of the calculated and the measured degradation curves of the inhibition control allows a more precise interpretation.

### Anaerobic bacteria inhibition test

There are two similar standardized anaerobic inhibition tests, OECD 224 and ISO 13461–1 and ISO 13461–2. These standards specify a screening method for assessing the potential toxicity of substances, mixtures, surface waters, groundwaters, wastewaters, effluents, sludges, or other environmental samples using digested sludge from anaerobic digestion reactors and by determining the production of biogas (carbon dioxide and methane) over a period of up to 3 days. The biogas production is measured by the determination of the overpressure in the test vessels. An inhibition can be calculated by comparing the gas production with a control vessel. The test is able to predict the maximum direct toxic effect and the time to reach this maximum. ISO 13641–1 specifies a general test, while ISO 13641–2 specifies a test for low biomass concentrations. The normal test duration is 48 h, the test temperature is at 35 °C, and 3,5-dichlorophenol is recommended as a reference substance. The mean EC_50_ determined in a ring test using (ISO 13461, OECD 224) was at 153 mg L^−1^. This test can also be used with wastewaters and it fits perfectly with a strategy of biotest systems for the evaluation of anaerobic biological treatment of wastewaters (Abwassertechnische Vereinigung ATV-AG 7.5.1 2004; Strotmann et al. 1993b).

## Bacterial inhibition tests for the soil compartment

Although the focus of this review is on aqueous systems, it should be mentioned that several bacterial toxicity tests also exist for soil compartments. Their objective is to evaluate potential toxic effects on soil microorganisms involved in biodegradation processes and nutrient cycles. Examples are the Nitrogen Transformation Test (OECD 216 2000) or the Carbon Transformation Test (OECD 217 2000), next to the nitrification inhibition tests according to ISO 15685 or the contact test for solid samples using the dehydrogenase activity of *Arthrobacter globiformis* (ISO 18187 2023). Another soil quality test estimates the decomposition of organic matter in contaminated soil (ISO 23265 2023).

## Non-standardized test systems

There also exist several non-standardized test systems used to determine bacterial toxicity. They include tests on cell membranes as well as on enzymes, tests based on the measurement of metabolites such as adenosintriphosphate (ATP) as well as microcalorimetric methods. Among these, the most widely tests used are enzymatic assays using dehydrogenases and the estimation of ATP luminescence as a measure of active biomass (Dalzell et al. 2002). The purposes range from the determination of toxic effects to the supervision of vital functions in living cells. For a general overview, see Bitton and Dutka (1986) and Dutka and Bitton (1986).

## Toxic effects on cell membranes and QSAR models

Organic compounds in the environment may be toxic for living organisms because they accumulate in and disrupt cell membranes and essential metabolic processes such as respiration and cell growth can be inhibited. The toxicity of such compounds often correlates with the logarithm of its partition coefficient between octanol and water (log P). Table 2 provides a summary of this correlation on the basis of the well-investigated chemical classes of (chloro)phenols and *n*-alkanols. Substances with a log *P* value between 1 and 5 are, in general, toxic for whole cells (Heipieper et al. 2007; Sikkema et al. 1995). Such chemicals, for example, toxic hydrocarbons, can only be degraded at a low rate. This needs to be taken into consideration for the design of biodegradation tests. This direct correlation is also used for quantitative structure–activity relationship (QSAR) models. Exceptions are compounds with very low water solubility (bioavailability) and those that are charged. Here, the Henderson-Hasselbalch-Equation can be used to calculate the percentage of non-charged substances for the QSAR model (Heipieper et al. 2007, 1994; Sikkema et al. 1995).

**Table 2:** LogP dependency of EC_50_ values (in mM and in mg L^−1^) of phenol/chlorophenols and *n*-alkanols. Data for growth inhibition test (EC_50_) with *Pseudomonas putida* (Heipieper and Martínez 2018)

Compound	LogP	EC_50_ (mM)	EC_50_ (mg L^−1^)
Phenol	1.45	8.6	809.35
4-Chlorophenol	2.40	2.0	257.12
2,4-Dichlorophenol	3.20	0.4	65.20
2,4,5-Trichlorophenol	4.05	0.08	15.80
2,3,4,5-Tetrachlorophenol	4.59	0.016	6.89
Pentachlorophenol	5.12	0.008	2.13
Methanol	− 0.76	1480.0	47419.20
Ethanol	− 0.28	345.0	15894.20
1-Butanol	0.88	30.1	2231.01
1-Hexanol	1.87	5.8	56.86
1-Octanol	2.92	1.1	8.46
1-Decanol	3.97	0.1	0.63

## Toxicity tests using enzymes

Toxicity tests with enzymes can be very reproducible, reliable, and often can be carried out easily. They include enzymes such as dehydrogenases, ATPases, esterases, phosphatases, urease, mixed function oxidases (MFO), aryl hydrocarbon hydroxylases (AHH), and peptidases (L-alanine-aminopeptidase, Dalzell et al. (2002)). For a detailed review, see Bitton and Dutka (1986) and Dutka and Bitton (1986). The test results from enzymatic tests can give information for possible toxic effects but they have the disadvantage that predictions to the real environment are restricted.

## Dehydrogenase test

The dehydrogenase test is frequently used for monitoring the biological activity of activated sludge (Bensaid et al. 2000; Pan et al. 2023) and also for the determination of toxicity of chemical compounds (Strotmann et al. 1993a, 1992). It is based on the fact that artificial acceptors take up the reduction equivalents produced in the microbial electron transport system (ETS) and are subsequently reduced. These reduced compounds form colored substances which can be analyzed spectrophotometrically. This system is easy to perform and can be used for various purposes, such as activated sludge (Bitton and Dutka 1986; Pan et al. 2023; Strotmann et al. 1993a, 1992; Yuan et al. 2019), anaerobic degradation and digestion processes (Wang et al. 2022), in soil (Järvan et al. 2014) and even for eukaryotic systems such as algae (Xie et al. 2008). Acceptor molecules for the reduction equivalents are TTC (2,3,5-triphenyltetrazolium chloride), INT (2-(*p*-iodophenyl)-3-(*p*-nitrophenyl)-5-phenyltetrazolium chloride), XXT (3′-[1-[(phenylamino)-carbonyl]-3,4-tetrazolium]-bis(4-methoxy-6-nitro) benzenesulfonic acid hydrate), resazurin (sodium salt of 7-hydroxy-3H-phenoxazin-3-on-10-oxid), and methylene blue (Liu 1983; Maurines-Carboneill et al. 1998; McCluskey et al. 2005; Strotmann et al. 1993a). It has to be mentioned that both methylene blue and TTC have a low affinity for electrons. Therefore, the tests using these indicators have to be carried out in the absence of oxygen. When using INT a time-consuming extraction step is necessary prior to spectrophotometry (Anderson et al. 1988). Resazurin has the advantage that no extraction step is necessary and is, therefore, a favorable method for activity determination and toxicity assessment (Liu 1981, 1983).

Concerning the estimation of activity, it was stated that there is not always a positive correlation between respiration activity and dehydrogenase activity in model wastewater treatment plants operated with municipal or industrial wastewater (Strotmann et al. 1993a). On the other hand, Pan et al. (2023) stated a positive correlation between respiration activity, dehydrogenase activity, and ATP content in a sequencing batch reactor (SBR) model using TTC and INT-based dehydrogenase tests. They measured the effects of heavy metal ions such as Cu^2+^, Cd^2+^, and Ni^2+^ and several antibiotics such as sulfamethoxazole, terramycine, and tetracycline. The reliability of the test system was demonstrated as well as the influence of pH activity of different phenolic compounds (Table S2). Furthermore, it could be shown in a shock loading experiment that the uncoupling agent 2.4-dinitrophenol inhibited the dehydrogenase activity whereas it enhanced the respiration activity as expected (Strotmann et al. 1993a). These results show that the dehydrogenase activity is a suitable test system for monitoring purposes as well as for toxicity assessment and can be used to provide additional information to the respiration inhibition test.

Up to now, the dehydrogenase test system has not been standardized with one exception aiming at soil quality, the contact test for solid samples using the dehydrogenase activity of *Arthrobacter globiformis* (ISO 18187 2023). It could be useful to standardize more variations of the dehydrogenase test in order to broaden the range of microbial toxicity tests for water and physiological parameters.

## Sensitivity of microbial toxicity tests

The bacteria toxicity tests presented here are used to determine the toxicity of chemical compounds and wastewaters. However, it has to be kept in mind, that the sensitivity of the different test systems may be different. The most sensitive test is the inhibition of nitrification activity, followed by the luminescent bacteria test. The respiration inhibition test and the growth inhibition test with sewage bacteria are less sensitive as well as the growth inhibition test with *Pseudomonas putida* and the dehydrogenase test (see Tables 3, 4, and S2). This assumption is in good accordance with data from the literature (Dalzell et al. 2002; Gutiérrez et al. 2002; Yuan et al. 2019). One has to take note that even standardized tests allow certain changes in its performance. For example, a prolongation of the test duration up to 27 h can increase the sensitivity of the respiration inhibition test (Gendig et al. 2003; ISO 8192 2007; ISO 9509 2006; Strotmann et al. 2020). Toxicity tests require effective reference substances in order to control the performance. Reliable results are usually obtained with chlorophenols, especially with 3,5-dichlorophenol. Ionic compounds such as Zn(II) and Cr(VI) give more variable results because physical–chemical interactions such as precipitation and adsorption may influence the test results (Dalzell et al. 2002; Gutiérrez et al. 2002; Strotmann et al. 2020). Mercury compounds would be ideal, but their use is no longer desirable due to environmental reasons.

**Table 3:** Toxicity of different phenolic compounds in different microbial toxicity tests (modified after Pagga and Strotmann 1999; Strotmann et al. 1994, 1993c). All concentrations are given in mg L^−1^

Test system	Respiration inhibition test (ISO 8192 2007)	Growth inhibition test (ISO 15522 1999)		Nitrification inhibition test (ISO 9509 2006)	Luminescent bacteria test (ISO 11348 2008)
Inoculum	Industrial sludge	Municipal sludge	Industrial sludge	Municipal nitrifying sludge	*Aliivibrio fischeri*
Phenol					
EC_20_	100	600	450	0.1	9
EC_50_	300	> 1000	880	1	29
EC_80_	800	> 1000	> 1000	2	250
3-Chlorophenol					
EC_20_	3	50	32	0.2	1
EC_50_	25	92	95	0.9	7
EC_80_	160	> 100	200	4	25
3,5-Dichlorophenol					
EC_20_	6	5	4	0.1	3
EC_50_	10	10	5	0.5	5
EC_80_	34	100	18	2	7
2,4-Dinitrophenol					
EC_20_	190	600	430	10	6
EC_50_	250	740	700	70	29
EC_80_	800	> 1000	> 1000	400	100

**Table 4:** Compilation of ecotoxicological data and bacterial toxicity data concerning 3,5-dichlorophenol

Test system	Test duration	EC_50_ or LC_50_ (mg L^−1^)	Parameter tested	Source
Algae				
*Pseudokirchneriella subcapitata (*= *Scenedesmus capricornutum)*	72 h	1.79–1.96	Growth inhibition	(Paixao et al. 2008)
	48 h	3.20–3.60	Growth inhibition	(Arensberg et al. 1995)
	96 h	2.30	Growth inhibition	(Shigeoka et al. 1988)
	72 h	3.38	Growth inhibition	(ISO 8692 2012)
*Desmodesmus subspicatus (*= *Scenedesmus subspicatus)*	72 h	5.00	Growth inhibition	Safety data sheet Sigma-Aldrich (2024)
	72 h	6.42	Growth inhibition	(ISO 8692 2012)
Invertebrates				
*Daphnia magna*	24 h	1.00–3.50	Reproduction	Safety data sheet Sigma-Aldrich (2024)
Fish				
*Crassius auratus*	2.5 h	2.99	Lethality	(Kishino and Kobayshi 1996)
	5 h	2.49	Lethality	(Kishino and Kobayshi 1996)
*Brachydanio rerio*	24 h	1.00–3.50	Lethality	(Devillers et al. 1985)
*Oryzias latipes*	96 h	2.30	Lethality	(Shigeoka et al. 1988)
*(*Smith et al. 1994*)*	96 h	3.50	Lethality	(Smith et al. 1994)
Bacteria				
Nitrifying activated sludge	≥ 4 h (ISO 9509); 30 min (ISO 8192)	4.60–5.60	Nitrification inhibition	(ISO 8192 2007; ISO 9509 2006)
*Aliivibrio fischeri*	5–15 min	3.52–3.58	Luminescence inhibition	(ISO 11348 2008; Strotmann et al. 2020)
Activated sludge	30 min or 180 min up to 27 h	20.3	Respiration inhibition	(ISO 8192 2007)
Sewage bacteria from activated sludge	4.5 to 6 h	8.1	Growth inhibition	(ISO 15522 1999)
*Pseudomonas putida*	16 h ± 1 h	21.4	Growth inhibition	(ISO 10712 1995)
Anaerobic sludge	48–72 h	153	Biogas production	(ISO 13461 2003)
Activated sludge	30 min	> 100	Dehydrogenase activity at pH 7.0	Not standardized

In the ECHA Guidance, the following order of increasing sensitivities is shown based on the publications of the German Federal Environment Agency UBA (1993), Reynolds et al. (1987), and Ren and Frymier (2003). The sensitivity increases from the respiration inhibition test to the inhibition control assays in biodegradation tests, over the growth inhibition test with *Pseudomonas putida* and as the most sensitive tests the inhibition of nitrification and the luminescent bacteria test. On the other hand, sensitivity is not the only criteria for selecting appropriate tests for assessing toxicity effects in WWTPs as mentioned above.

When comparing microbial toxicity tests with classical ecotoxicological tests (which are also designated as ecotoxicity tests) such as the microalgae test with *Pseudokirchneriella subcapitata (*= *Scenedesmus capricornutum)* and *Desmodesmus subspicatus (*= *Scenedesmus subspicatus)*, the Daphnia test with *Daphnia magna* and the fish test with *Crassius auratus*, *Brachydanio rerio*, *Oryzias latipes*, *and Platichthys flesus*, it is striking that only the sensitivity of the luminescent bacteria test and the nitrification inhibition test is in the same range (see Table 4). Also, in other studies, a good correlation of the data in these test systems has been published (Bringmann and Kühn 1980; Hernando et al. 2003, 2005; Shigeoka et al. 1988). Therefore, the microbial test systems may be suitable as range finders or pre-tests for other ecotoxicological tests with algae, invertebrates, and fish species. This is also beneficial as animal welfare problems are avoided when using microbial toxicity tests. Therefore, the strict separation of microbial toxicity tests from classical ecotoxicological tests systems should be overthought. It also has to be mentioned that sometimes microbial toxicity tests are seen as a part of ecotoxicological tests and sometimes are seen separately. Nevertheless, microbial test systems could be integrated as a useful help in a toolbox or a test battery containing microbial as well as ecotoxicological tests for the determination of toxicity of chemical compounds and for monitoring purposes (Barcelo et al. 2020; Farré and Barcelo 2003).

## A promising integrated toolbox: microbial and ecotoxicity tests in combination with biodegradation tests

**Fig. 3:**
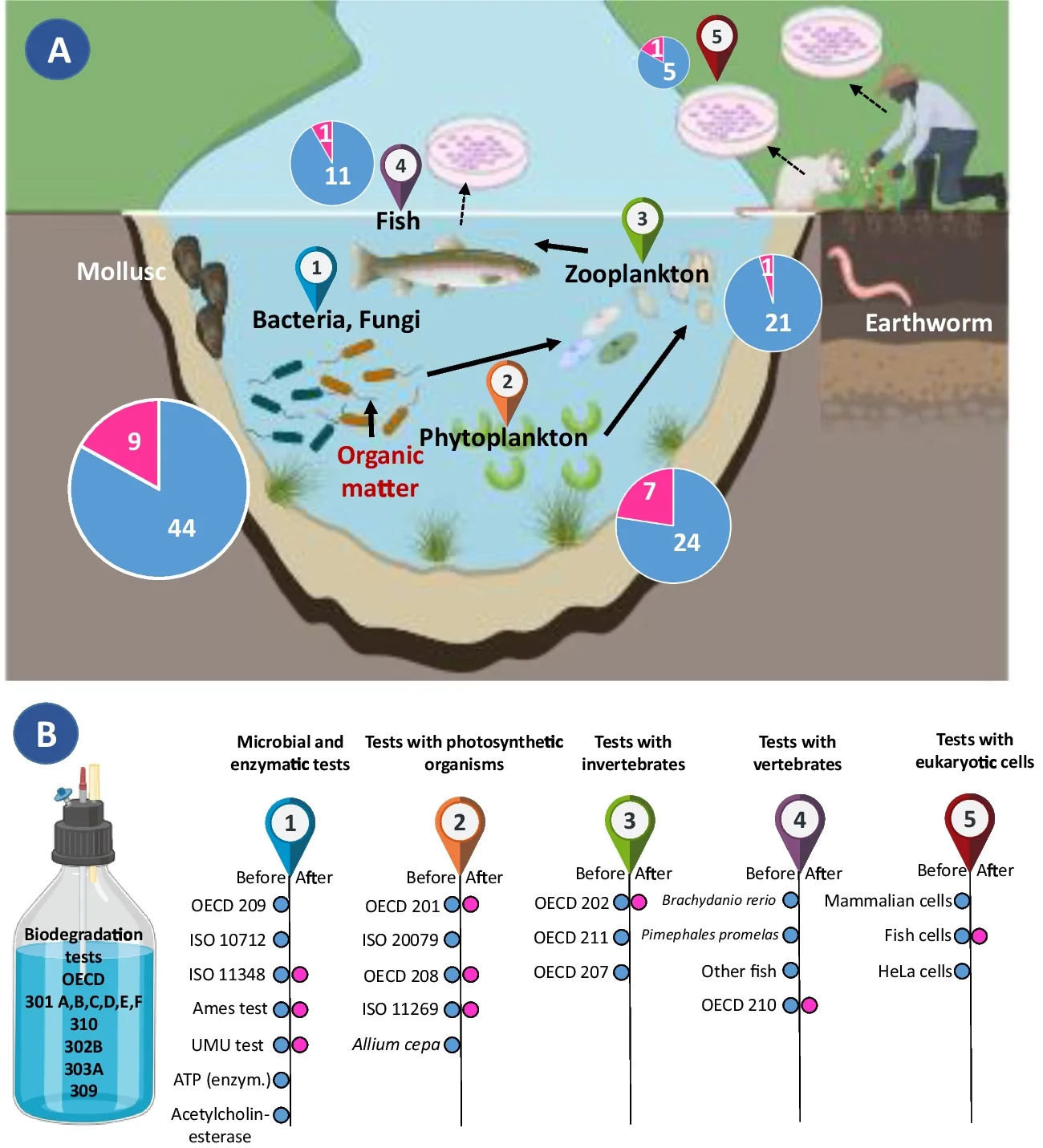
Ecotoxicity tests used before (blue) or after (pink) standardized biodegradation tests. **A** Schematic trophic chain showing the position of each family of ecotoxicity tests selected by the 39 publications identified in Table S4. The pie charts inside indicate the times the tests were used in the publications. **B** Details of the ecotoxicity tests performed either before or/and after OECD standard biodegradation tests. The Ames test and the UMU test are tests for mutagenicity. (ATP: adenosine triphosphate)

In order to properly characterize single chemical compounds and mixtures of chemicals a number of studies have been performed where microbial toxicity tests, ecotoxicity tests, and OECD biodegradation tests have been combined. A bibliometric analysis from two databases (Science Direct and PubMed) was carried out in order to analyze studies integrating toxicity tests when assessing the biodegradation of molecules or mixtures of compounds. Figure S1 shows that 74 articles were retained after selection using specific key words. From these 74 articles only 39 studies used OECD biodegradation tests. Table S3 gives a detailed analysis of the biodegradation tests carried out as well as the associated ecotoxicity tests. The main information that emerges shows that microbial and ecotoxicity tests are generally performed on pure compounds or mixtures before biodegradation tests, whereas only few studies (8/40) focus on the possible toxicity of metabolites. A summary of the data analysis is given in Fig. 3. In addition, this figure also illustrates that there is a close connection between microbial toxicity, biodegradation, and classical ecotoxicology, but it also has to be mentioned that ecotoxicological details are beyond the purpose of this review. Although a wide variety of ecotoxicity tests were carried out, the majority concerned microbial toxicity. Most studies (25/39) combined the luminescent bacteria toxicity test using *Aliivibrio fischeri* with biodegradation tests. This choice is primarily based on the ease of the test set up than on its ecological relevance. In order to assess the ecotoxicological impact of a compound or mixture, it is necessary to combine tests using organisms from different trophic levels. 6 of the 39 studies combined the impact of the compound on a microorganism, a photosynthetic organism, and an invertebrate (*Daphnia magna*) (Gartiser et al. 2009; Pedrazzani et al. 2012; Stolte et al. 2012; Sagi et al. 2018; Gatidou et al. 2021). Finally, four studies include mutagenicity or genotoxicity tests in order to assess the impact of leachate of materials, effluents (Gartiser et al. 2009, 2017), detergents (Padrazzani et al. 2012), or by-products of antibiotics due to photodegradation (Bergheim et al. 2015). Therefore, it is recommended that the ecotoxicity assay implemented with biodegradation studies should consider the ecosystem affected by the release of the chemical compounds.

## The interplay of microbial toxicity and biodegradation

**Fig. 4:**
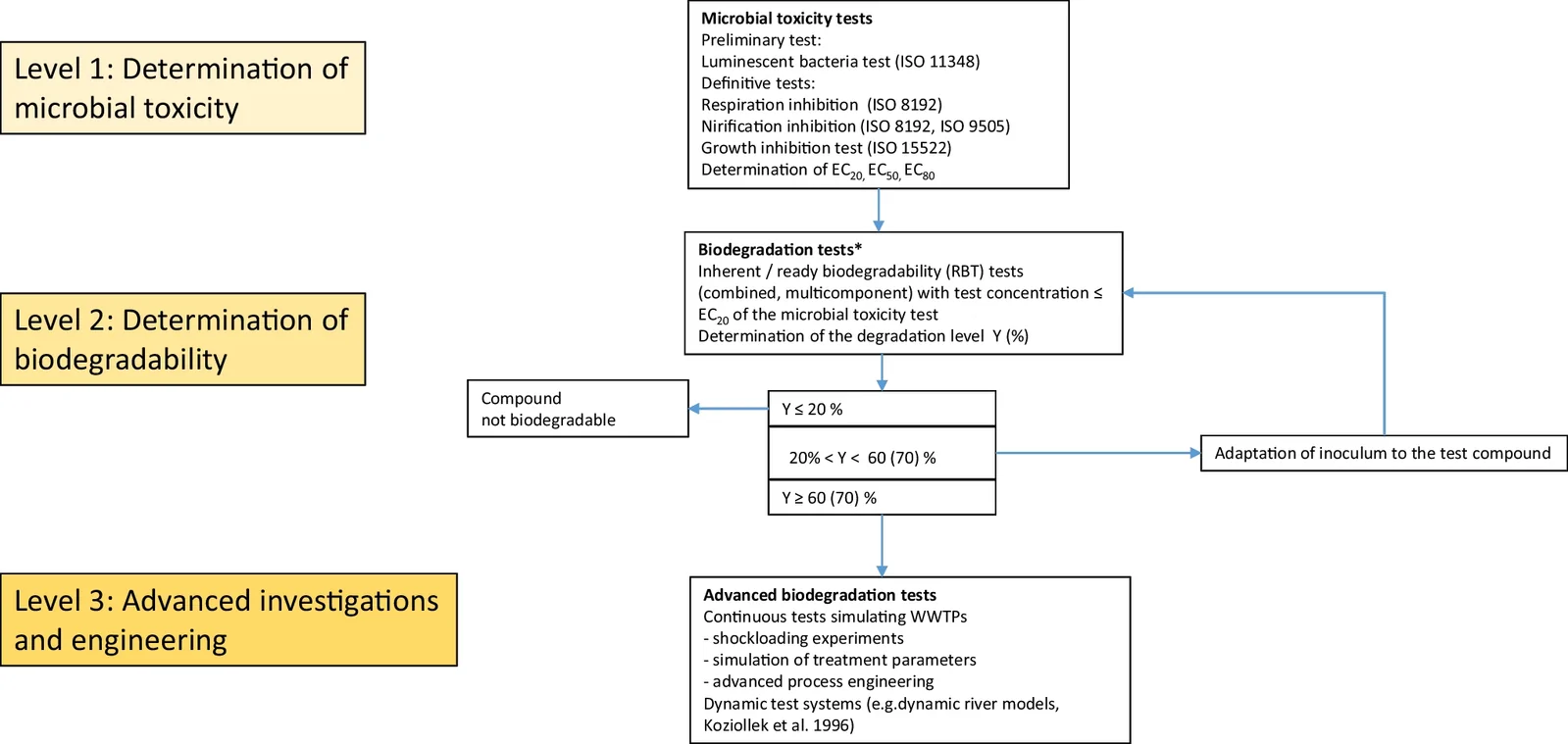
Flow chart illustrating the interconnection of microbial toxicity tests, ready biodegradation tests (OECD 301 A to F 1992), inherent biodegradation tests (OECD 302 A 1981, OECD 302 B 1992, OECD 302 C 1981, OECD 304 A 1981) and advanced engineering techniques

It is obvious that toxic effects of chemicals can severely affect biodegradation processes and also hinder the adaptation of bacteria to degrade xenobiotic compounds. This may be due to the original substance, by metabolic products which are formed during the biodegradation process, or by a shift of the pH value. For example, during the degradation of 1,2-dichloroethane the metabolites 2-chloroethanol and chloroacetaldehyde are formed, the latter being well known to be rather toxic and mutagenic (Dijk et al. 2003; Janssen et al. 1984, 1995; Strotmann et al. 1990; Strotmann and Röschenthaler 1987). Many bacteria are able to handle these restrictions and degrade not only toxic substances but also toxic metabolites. This may be due to genetic processes by induction of degrading enzymes or by the excretion of toxic metabolites. These enzymatic processes are often designated as detoxification reactions and can include different types of reactions such as dehalogenation, hydrolysis, hydroxylation, dealkylation, reductions of nitro groups, deamination, ether cleavages, conversion of nitriles to amides, and conjugations (Alexander 1994). Besides these enzymatic reactions, also the cultivation conditions of bacteria can positively affect the biodegradation and lower toxic effects. This could be demonstrated when *Pseudomonas putida* US2 was immobilized and used to degrade 2-chloroethanol which is a metabolite in the degradation pathway of 1,2-dichloroethane. During the biodegradation process, a liberation of protons occurs during a dehalogenation process, which lowers the pH value. It could be demonstrated that the way of immobilization and an effective pH control together with a supplementation with a secondary substrate had a significant influence on the biodegradation efficiency leading to a technical application in a bioreactor (Knippschild and Rehm 1995; Overmeyer and Rehm 1995). Concerning microbial toxicity 2-chloroethanol exhibits a rather low toxicity with an EC_50_ at 6 g L^−1^ (74.52 mM) in the luminescent bacteria test (30 and 60 min test, unpublished results). In an OECD 301F biodegradation test 2-chloroethanol could be degraded to an extent of 93% after a 5 to 6 days lag period which is a clear indication for adaptation processes (Reuschenbach et al. 2003). These results show that a low microbial toxicity often positively correlates with a high adaptation potential of the inoculum in a biodegradation test. This is possible because the tolerant species whose degradation potential is already present prevail. Concerning the cyclic nitrogen-containing compound morpholine similar results could be obtained. Here also a low microbial toxicity (15% inhibition at a concentration of 1000 mg L^−1^ in a respiration inhibition test with not adapted activated sludge, 5% inhibition at a concentration of 100 mg L^−1^ in a nitrification inhibition test) seems to be a prerequisite for a successful adaptation process. Morpholine showed a 16 days lag period before the onset of biodegradation in an OECD 301F test system. The final extent of biodegradation was rather high at 87 to 89%. Further extended shock loading experiments with morpholine in a continuously operated laboratory scale WWTPs confirmed these results (Strotmann et al. 1993c). All in all, these examples show that a low microbial toxicity is very favorable to adapt inocula to a xenobiotic compound and stress the importance of microbial toxicity tests as pre-tests for biodegradability tests. This fact is also illustrated in Fig. 4 where the close interplay of microbial toxicity tests and biodegradation tests is illustrated which can also have consequences for subsequent engineering and technical implementations.

The objective of biodegradability testing is not only to conclude whether a test substance is biodegraded or not, but also to indicate whether biodegradability is an intrinsic substance property. Inhibition of the inoculum is regarded as a disturbance of possible biodegradation processes and should be avoided within biodegradation tests. Therefore, Annex II of OECD 301 1992 (see also OECD 301 A to F 1992) recommends that substances suspected to be toxic to the inoculum should preferably be tested at concentrations corresponding to 1/10 of the EC_50_ values obtained in the activated sludge respiration inhibition tests (OECD 209 2010). ISO 10634 2018 provides a guidance for the preparation and treatment of poorly water-soluble organic compounds for the subsequent evaluation of their biodegradability. Here, it is mentioned that by adding an inert carrier material the bioavailability of a test substance may be reduced due to adsorption processes. In this way, possible toxic effects of the test substance to microorganisms may be reduced and an increase of biodegradation is possible. On the other hand, the limited bioavailability of the test substance due to adsorption may also result in a decrease of biodegradation. The reason lies in the limited access of the test substance to the inoculum. Only few publications addressing these controversial effects are available (Gartiser et al. 2023; Nabeoka et al. 2020; Timmer et al. 2019; van Ginkel et al. 2008). The ECHA guidance (2023) states that inhibitory substances could be tested with the lowest test substance concentration possible, which may be achieved using the Closed Bottle Test (OECD 301 D 1992). Unfortunately, the inoculum concentration and the degradation potential are very low in this test and thus counteracting the desired effect. Inhibitory effects on the inoculum in biodegradability test may often be manifested by longer lag-periods, which is obvious by achieving the 10% level. Another option to avoid the influence of toxic effects is the use of several concentrations of the test substance (e.g., 10 and 20 mg L^−1^ TOC in the OECD 301 B 1992 test or 50 and 100 mg L^−1^ ThOD in the OECD 301 F 1992 test). All in all, there exist no general rules to reduce inhibitory effects in biodegradation tests. Therefore, the best option is a try and error approach by practical testing.

## Characterization of inocula for biodegradation tests and microbial toxicity tests

### General considerations

Bacterial inocula play an important role in biodegradation and bacterial toxicity tests. Therefore, it may be helpful to characterize the quality of the inocula used. This can be done by genetic analysis of the bacteria present in the inoculum, but this is often difficult and time-consuming (Forney et al. 2001; Muñoz-Palazon et al. 2018; Prosser et al. 2007; Saunders et al. 2016; Wu et al. 2019; Xia et al. 2010; Zhang et al. 2012). This approach is very promising for scientific reasons, but it will probably not fully describe the physiological properties and degradation capacities of inocula required for practical microbial toxicity and biodegradation tests.

The inocula used in biodegradation tests mainly originate from municipal WWTPs whose microbial composition may be identified to a certain degree and contain a common core of bacteria which can be found in all these plants (Fig. 1) (Brown et al. 2019b, 2019a; Madoni 2011; Maza-Marquez et al. 2018). If toxicity tests are carried out with pure strains, they cannot be representative for the whole biocenosis in natural or technical systems or they are even entirely missing. For example, the marine bacterium *Aliivibrio fischeri* used in the luminescent bacteria test is not present in WWTPs. Furthermore, a lack of reproducibility of standardized biodegradation tests has been described, which was associated with the difficult control of the inoculum by terms of density and diversity (Davenport et al. 2022; Strotmann et al. 2023; Thouand et al. 2011). However, investigations have shown that it is possible to sufficiently characterize an inoculum before performing a biodegradation test or a toxicity test. The aim is to limit or control this variability by adjusting both the amount and the activity of the inoculum. There are three main current techniques covering three levels of information. The first one is the measurement of cell density consisting of microscopic observation, the determination of the cell biomass (dry matter, optical density, and other methods) (Ahtiainen et al. 2003; Struijs et al. 1995; Thouand and Block 1993; Thouand et al. 1995), the measurement of cell density by optical means (Riedel et al. 2023; Thouand et al. 1995), the cell counting (total and viable cells, cytometry) (Brown et al. 2019a; Goodhead et al. 2014; Thouand and Block 1993; Thouand et al. 1995) and cultivation methods (colony forming units, CFU and most probable number, MPN) (Gartiser et al. 2023; Thouand and Block 1993; Thouand et al. 1995; Vázquez-Rodríguez et al. 2007). The second group consists of phenotyping methods such as the determination of activity levels (ATP, reduction of tetrazolium salts whose transformation state is an activity indicator) (Pan et al. 2023; Thouand and Block 1993; Vázquez-Rodríguez et al. 2007) and physiological profiling (BIOLOG) (Guo et al. 2010; O’Malley 2006; Vázquez-Rodríguez et al. 2007). For example, O’Malley et al. (2006) selected a better inoculum for an OECD 301 1992 test by using BIOLOG GN microplates by comparing their whole community carbon source utilization profile. Now it is even possible to enlarge the comparison by using the Phenotype Microarrays (BIOLOG) with 190 Carbon sources, 95 nitrogen sources, 59 phosphorous sources, and 35 sulfur sources (personal communication). The third group compiles methods to identify cellular diversity such as genotyping and metagenomic methods (denaturing gel electrophoresis, DGGE; 16 s and 18 s rRNA analysis and shotgun methods in order to identify the main microbial communities present. Together with metatranscriptomic, metaproteonomic, and metabolomic methods, this level is more difficult to achieve and only a few publications associated with the evaluation of the biodegradation of substances have used it Goodhead et al. 2014; Poursat et al. 2020). Of all these characterization methods, only the measurement of dry matter is suggested in the standards. We believe that it is now possible to increase the information on the inoculum before carrying out a biodegradation test, at least by using more descriptors. At a minimum dry matter, cell counting and a physiological profile (using the physiological potential of an inoculum, PPI (see below) and BIOLOG) should be used, for this would allow a better comparison between tests or to identify misfunctioning inoculum before starting a biodegradation test.

## The physiological potential of an inoculum (PPI): a new integrative concept of inocula characterization based on biodegradation and microbial toxicity test data

Besides the general physiological description of the inoculum with the help of the BIOLOG system also a specific system can be used which is directly aiming at OECD/ISO normalized biodegradation test. Concerning the physiological biodegradation-oriented capacities there are two approaches which can be applied. One is based on the estimation of the biodegradation adaptation potential (BAP) and the other on the resistance of an inoculum to defined toxic compounds which can be designated as a chemical resistance potential (CRP). Therefore, a successful inoculum should meet certain defined criteria concerning the BAP and the CRP. As these two parameters concern the physiology of degrading bacteria they can be combined to a new parameter, the physiological potential of an inoculum (PPI), which can be regarded as a tool to estimate the quality of an inoculum for biodegradation tests.

The BAP is obtained by the determination of the lag period in a biodegradation test with a defined test chemical, which is the period before degradation takes obviously place. It may describe as well the ability of an inoculum to adapt to an unknown compound. BAP tests can be performed with certain chemical compounds, which can only be degraded after a certain lag period during which the bacteria acquire the capability to degrade these compounds. Examples for such compounds are morpholine, NTA, ibuprofen (adaptation periods in the range of 9 to 16 days), 2-chloroethanol, and diethylene glycol (adaptation periods 5 to 8 days). In general, different compounds require certain adaptation capabilities. NTA, morpholine, and ibuprofen are compounds which require a high adaptation potential, whereas 2-chloroethanol and diethylene glycol require a medium adaptation potential. Compounds such as acetate, glycerol, and benzoate (adaptation periods 0 to 1 day) are not useful because they need only a very short time and require a low adaptation potential. Performing such tests, the adaptation potential of an inoculum can be exactly examined and the inoculum characterized. Adaptability can be grouped into three classes, where an inoculum of class 1 (lag period 0 to 2 days) has the lowest adaptation potential, an inoculum of class 2 (lag period > 2 days to 5 days) has a medium adaptation potential, and an inoculum of class 3 (lag period > 5 days) the highest. In all tests, the extent of biodegradation for ready biodegradability (ranging from 60 to > 80%, depending on the test system used) has to be met. It is important to perform these tests in a way that inocula are given the chance to reach the highest-class level. Inocula with a BAP of class 2 to class 3 are assumed to perform better in a biodegradation test than inocula with a BAP of class 1. Therefore, these inocula meet certain quality criteria concerning adaptability and biodegradation patterns. Details to this new concept were described before (Strotmann et al. 2023).

Besides the adaptation potential of an inoculum also the resistance of an inoculum to a toxic reference compound can be used to characterize it in terms of physiological properties and can be designated as chemical resistance potential (CRP). The CRP is based on the determination of an EC_50_ in bacterial toxicity tests with a well-defined reference compound. Ideally, an inoculum should show a certain resistance against inhibitory compounds. On the other hand, the resistance should not be too distinct. The concept is primarily aiming at inocula from WWTPs. Here the inoculum should be tested in a respiration inhibition test (heterotrophic respiration) and also in a nitrification inhibition test (autotrophic respiration) using for example 3,5-dichlorophenol which is a well-characterized toxic compound. These two test systems are up to now the most relevant test systems to characterize the physiological activity of activated sludge from a WWTP (Yuan et al. 2019). Therefore, a scheme was developed to characterize activated sludge using these test systems. There are different classes concerning the CRP. Class 1 represents a low chemical resistance, class 2 a moderate resistance, and class 3 a high resistance to 3,5-dichlorophenol. Details of the proposed scheme are shown in Table 5.

**Table 5:** Estimation of the physiological potential of an inoculum (PPI) based on the biodegradation adaptation potential (BAP) and the chemical resistance potential (CRP) based on respiration and nitrification inhibition tests (modified and extended after Strotmann et al. (2023)

BAP (biodegradation adaptation potential)			
	Test system	Extent of biodegradation (%)	Lag period (days)
*Class 1 (low adaptation potential)*			
Benzoate, acetate, glycerol, cyclohexanone, 2-ethylacrylate	OECD 301 F 1992	58–96	0–1
*Class 2 (medium adaptation potential)*			
Aniline, acrylic acid, phenol, 1,5-pentanediol, 4-isopropylphenol	OECD 301 F 1992	70–94	2.3–5
*Class 3 (high adaptation potential)*			
NTA, morpholine, 2-chloroethanol, diethylene glycol, ibuprofen, 4-fluorophenol	OECD 301 A/D 1992, OECD 301 F 1992	62–98	6–16
CRP (Chemical resistance potential)			
	Test system	EC_50_ (mg L^−1^)	
*Class 1 (low resistance potential)*			
3,5-Dichlorophenol	Respiration inhibition (ISO 8192 2007)	< 5	
	Nitrification inhibition (ISO 9509 2006)	< 0.1	
*Class 2 (medium resistance potential)*			
3,5-Dichlorophenol	Respiration inhibition (ISO 8192 2007)	5–20	
	Nitrification inhibition (ISO 9509 2006)	0.1–6	
*Class 3 (high resistance potential)*			
3,5-Dichlorophenol	Respiration inhibition (ISO 8192 2007)	> 20	
	Nitrification inhibition (ISO 9509 2006)	> 6	
Physiological potential of inoculum (PPI, combination of BAP and CRP)			
	BAP	CRP	
Very high*	Class 3	Class 3	
High**	Class 3	Class 2	
Medium**	Class 2	Class 2 or 3	
Low ***	Class 1	All classes	

^*^Highly specialized inoculum. Maybe not suitable for prediction of biodegradability under natural conditions

^**^Ideal inoculum for prediction of biodegradation under natural conditions

^***^Inoculum not recommended for biodegradation tests due to low BAP

A combination of the BAP and CRP of an inoculum would be ideal to characterize the intrinsic physiological potential of inocula (PPI) for biodegradation tests concerning either the adaptation capabilities and the chemical resistance potential. Therefore, quality groups for the physiological potential of an inoculum ranging from a very high to a low physiological potential have to be met (Table 5). An ideal inoculum for biodegradation tests should meet the criteria for a high or medium physiological potential. A very high potential of an inoculum would mean that the bacteria are extremely well adapted. Such an inoculum would not be representative for a realistic estimation of biodegradability under natural conditions. On the other hand, a low PPI would mean that the inoculum would be unsuitable and would deliver unrealistic results in a biodegradation test. Together with the genetic analysis of the inoculum, this concept with physiological parameters might be ideal for further future developments.

## A technical application: bacterial toxicity tests for monitoring wastewater treatment plants

### Laboratory tests

The use of bacterial toxicity tests is not restricted to characterize chemical compounds or important information for biodegradation tests. It can also be used for monitoring purposes. There are several fields of application for WWTPs, and if aqueous solutions or extracts are available, also for composting plants, soil, and landfills.

The easiest, fastest, and most cost-effective method is the use of the short-term respiration test (ISO 8192 2007). The test performance is the same as described for chemical compounds. Practice showed that the information obtained is sufficient to predict and, therefore, prevent toxic effects on activated sludge in a treatment plant. In order to be able to estimate the usefulness of laboratory tests, they should be compared with studies in technical plants or continuous run test facilities. Extensive investigations showed that the EC_20_ obtained in short-term respiration tests represents a very safe limit for the prediction of toxic effects by contaminated sewage. The effluent quality does not deteriorate if the EC_20_ is not exceeded. The results show as well, that in the case of long-term exposure, mixed populations may react by adaptation or degradation of toxic substances and thereby reduce the initial toxic effects (Pagga 1985, 1986; Pagga and Günthner 1981; Pagga et al. 1986; Strotmann and Weisbrodt 1994). Toxic effects that are not directly related to the respiration of the activated sludge such as the disruption of flocculation, may still worsen the effluent quality, but cannot be detected using respirometric methods.

According to the ECHA Guidance for assessing potential toxic effects on microorganisms in WWTPs, the preferred tests are those using a mixed inoculum because these tests assess the performance of the entire microbial community. For this purpose, the activated sludge respiration inhibition test (OECD 209 2010) is the most important test. The nitrification inhibition test (ISO 9509 2006; ISO 15685 2012) covers the performance of nitrifying organisms. Single species tests such as the cell multiplication inhibition test with *Pseudomonas putida* or tests with *Pseudomonas fluorescens* or *Escherichia coli* have a lower relevance for WWTPs (ECHA 2023). It should be noted, that there are also protozoa toxicity tests based for example on ciliates for assessing inhibitory effects on activated sludge, but this is beyond the objectives of this review paper.

## The BASF toximeter as a continuous monitoring system

One of the largest biological WWTP worldwide is being operated by BASF SE in Ludwigshafen Germany. The basic data of this plant are summarized in Table S4. The influent, the mixture of all wastewaters of the chemical sites and of two towns, is continuously monitored by a so-called toximeter which is based on continuous respiration measurements.

Basically, this toximeter is a miniaturized wastewater treatment plant with a primary clarification tank, an aeration tank, and a secondary clarification tank, which is operated in the same way as the real technical plant. However, the location is on the factory premises, about 3.5 km away from the WWTP, which results in a response time of around half an hour. In this time, appropriate measures can be taken in case of a toxic load of the wastewater, e.g., the temporal diverting of the wastewater into a collecting basin.

The respiration rate of the activated sludge in the toximeter is assumed to be the same as in the technical plant. It is monitored by a discontinuous intermittent aeration of the aeration tank. If a maximum oxygen concentration of about 3 mg L^−1^ is reached, the aeration is switched off. The bacteria continue to respire and the oxygen concentration drops. When a minimum value of approximately 0.5 mg L^−1^ is reached, the aeration process restarts again automatically. In the oxygen depletion phase, the respiration rate is calculated and graphically indicated. A decreasing respiration rate can reliably indicate a toxic shock load, which may damage the activated sludge. Due to the intoxication of the sludge, it is no longer possible to indicate the end of a toxic influent. Therefore, a second device, the toxicontrol unit, is operated simultaneously, which is continuously run with fresh uncontaminated activated sludge from a separate storage unit.

In addition to the respiration measurement, also the dissolved organic carbon (DOC) and other relevant substances are continuously measured in the influent and effluent. The treatment performance determined by the DOC decrease is an additional important criterion for the safe operation of the monitoring plant.

For many years the operation of the respiration-based toximeter provided a safe monitoring, but in the spring of 1984, a serious disruption occurred in the wastewater treatment plant with massive negative consequences for the effluent quality. The treatment process suddenly collapsed and the untreated wastewater was discharged for a certain time directly into the river Rhine. After a few hours, the activated sludge recovered again and its activity proved to be even better than before. The question arose why the toximeter did not warn the operators in good time. Surprisingly, no inhibition of respiration was measured, but, in contrast, a significant stimulation of the respiration activity was observed. Therefore, the aeration in the plant proved to be too weak to provide enough air to treat the influent. There had been no unusual discharges of wastewaters and analytical measurements showed no irregularities in the influent. Subsequent studies such as simulation tests with uncoupling agents like 2,4-dinitrophenol and other different pollutants could not clarify the situation. Therefore, two basic points can be derived from this incident. Firstly, it can be concluded that respiration is an important monitoring parameter, but that not all incidents can be detected with it. A battery of different additional microbial toxicity tests (e.g., nitrification and dehydrogenase activity for an additional monitoring of the activated sludge activity and the luminescent bacteria test for monitoring the influent and effluent) would be useful efforts to detect certain incidents. Secondly, not only a respiration inhibition can cause trouble, but also an unexpected increase of the respiration rate. Therefore, respiration has to be maintained in a certain safe interval, and leaving this interval can be regarded as an incident. But not only chemical substances could have been the reason, but also changes of the biocenosis of the activated sludge organisms. From today’s perspective, after the devastating impact of Corona on the health of humanity, one could also consider the role of bacteriophages, which can damage bacteria. Bacteriophages are present in activated sludge in high concentrations (see Fig. 1). These viruses could have caused a stimulation of metabolic activity of the activated sludge bacteria, something like an “euphorization.” Consequently, this might have caused the proliferation of certain bacterial species that suddenly started to grow much faster and outgrow the other species present. The result was a huge increase in metabolic activity and an enormous need for oxygen, which could no more be supplied. All these factors may have led to a temporary overload of the system and, as a consequence, a decrease of the effluent quality.

## Conclusions and future aspects

In this review, the most important microbial toxicity tests which have been normalized by the OECD and ISO are presented. The differences concerning the sensitivity of the test systems and the environmental compartments which they are aiming at are discussed as well as their advantages and disadvantages for a certain use. There is no single test which meets all requirements. It was also shown how important bacterial toxicity tests are for biodegradation testing. Therefore, an integrated concept which includes both, microbial toxicity tests and adequate biodegradation tests is helpful. The concept of the physiological potential of an inoculum (PPI) based on the biodegradation adaptation potential (BAP) and the chemical resistance potential (CRP) may be useful for such a concept. Furthermore, the set-up of a toolbox also containing certain ecotoxicological tests should be considered and might be useful for the near future. But there are two more points of interest concerning future testing. The first one is the potential which lies in the different fields dealing with “omics” (genomics, proteomics, metabolomics, metagenomics) which will gain increasing importance with the further development of modern technology and information processing. Undoubtedly, these technological improvements will also influence the existing norms and also the subsequent regulatory aspects. The second aspect concerns the field of artificial intelligence. In the field of biodegradation, there are successful projects which have been launched and which aim at predicting biodegradation on the basis of chemical structures and already existing or new test results. Artificial intelligence will certainly gain an increasing practical importance also in this field. Therefore, in the near future new insights will certainly arise which will open much more interesting and important aspects and pathing the way for promising future developments.

## Supplementary Information

Below is the link to the electronic supplementary material.

Supplementary file1 (PDF 310 KB)

## Data Availability

All data generated or analyzed during this study are included in this published article (and its supplementary information files).

## References

[CR1] Abbas M, Adil M, Ehtisham-ul-Haque S, Munir B, Yameen M, Ghaffar A, Abbas Shar G, Tahir MA, Iqbal M (2018) *Vibrio fischeri* bioluminescence inhibition assay for ecotoxicity assessment: a review. Sci Total Environ 626:1295–1309. 10.1016/j.scitotenv.2018.01.066 29898537 10.1016/j.scitotenv.2018.01.066

[CR2] Abwassertechnische Vereinigung ATV-AG 7.5.1 (2004) Anaerobe Testverfahren zu Abbaubarkeit, Hemmung und Aktivität. Korrespondenz Abwasser 51(9):997–1002

[CR3] Ahtiainen J, Aalto M, Pessala P (2003) Biodegradation of chemicals in a standardized test and in environmental conditions. Chemosphere 51(6):529–537. 10.1016/S0045-6535(02)00861-5 12615106 10.1016/S0045-6535(02)00861-5

[CR4] Alexander M (1994) Biodegradation and bioremediation. Academic Press, San Diego

[CR5] Anderson K, Koopman B, Britton G (1988) Evaluation of int-dehydrogenase assay for heavy-metal inhibition of activated sludge. Water Res 22:349–353. 10.1016/S0043-1354(88)90220-5

[CR6] Araujo CV, Nascimento RB, Oliveira CA, Strotmann UJ, da Silva EM (2005) The use of Microtox to assess toxicity removal of industrial effluents from the industrial district of Camacari (BA, Brazil). Chemosphere 58(9):1277–1281. 10.1016/j.chemosphere.2004.10.036 15667847 10.1016/j.chemosphere.2004.10.036

[CR7] Arensberg P, Hemmingsen VH, Nyholm N (1995) A miniscale algal toxicity test. Chemosphere 30(11):2103–2115. 10.1016/0045-6535(95)00090-U

[CR8] Bain PA, Williams M, Kumar A (2014) Assessment of multiple hormonal activities in wastewater at different stages of treatment. Environ Toxicol Chem 33:2297–2307. 10.1002/ETC.2676 24975364 10.1002/etc.2676

[CR9] Barcelo D, Zonja B, Ginebreda A (2020) Toxicity tests in wastewater and drinking water treatment processes: a complementary assessment tool to be on your radar. J Environ Chem Eng 8:104262. 10.1016/j.jece.2020.104262

[CR10] Bensaid A, Thierie J, Penninckx M (2000) The use of the tetrazolium salt XTT for the estimation of biological activity of activated sludge cultivated under steady-state andtransient regimes. J Microbiol Methods 40:255–263. 10.1016/s0167-7012(00)00130-5 10802142 10.1016/s0167-7012(00)00130-5

[CR11] Bergheim M, Gminski R, Spangenberg B, Debiak M, Bürkle A, Mersch-Sundermann V, Kümmerer K, Gieré R (2015) Antibiotics and sweeteners in the aquatic evironment: biodegradability, formation of phototransformation products and in vitro toxicity Eviron Sci Pollut Res 22:18017–18030 10.1007/s11356-015-4831-x 26169816

[CR12] Bertanza G, Papa M, Pedrazzani R, Repice C, Mazzoleni G, Steimberg N, Feretti D, Ceretti E, Zerbini I (2013) EDCs, estrogenicity and genotoxicity reduction in a mixed (domestic+textile) secondary effluent by means of ozonation: a full-scale experience. Sci Total Environ 458–460:160–168. 10.1016/j.scitotenv.2013.03.108 23648445 10.1016/j.scitotenv.2013.03.108

[CR13] Bertanza G, Steimberg N, Pedrazzani R, Boniotti J, Ceretti E, Mazzoleni G, Menghini M, Urani C, Zerbini I, Feretti D (2022) Wastewater toxicity removal: integrated chemical and effect-based monitoring of full-scale conventional activated sludge and membrane bioreactor plants. Sci Total Environ 851:158071. 10.1016/j.scitotenv.2022.158071 35988629 10.1016/j.scitotenv.2022.158071

[CR14] Bitton G, Dutka BJ (1986) Toxicity testing using microorganisms, Vol. 1. CRC Press, Boca Raton

[CR15] Bringmann G, Kühn R (1980) Comparison of the toxicity threshold of water pollutants to bacteria, algae and protouoa in the cell multiplication inhibition test. Water Res 14(3):231–241. 10.1016/0043-1354(80)90093-7

[CR16] Brown MR, Baptista JC, Lunn M, Swan DL, Smith SJ, Davenport RJ, Allen BD, Sloan WT, Curtis TP (2019a) Coupled virus - bacteria interactions and ecosystem function in an engineered microbial system. Water Res 152:264–273. 10.1016/j.watres.2019.01.003 30682570 10.1016/j.watres.2019.01.003

[CR17] Brown MR, Hands CL, Coello-Garcia T, Sani BS, Ott AIG, Smith SJ, Davenport RJA (2019b) Flow cytometry method for bacterial quantification and biomass estimates in activated sludge. J Microbiol Methods 160:73–83. 10.1016/j.mimet.2019.03.022 30926316 10.1016/j.mimet.2019.03.022

[CR18] Carvalho AR, Pérez-Pereira AI, Couto CMC, Tiritan ME, Ribeiro CMR (2022) Assessment of effluents quality through ecotoxicological assays: evaluation of three wastewater treatment plants with different technologies. Environ Sci Pollut Res 29:963–976. 10.1007/S11356-021-15671-Y/TABLES/3 10.1007/s11356-021-15671-y 34345989

[CR19] CEFIC (2023) European chemical industry council - facts and figures. https://cefic.org/app/uploads/2023/12/2023_Facts_and_Figures_The_Leaflet.pdf. Accessed 25 Aug 2024

[CR20] Cui D, Kong L, Wang Y, Zhu Y, Zhang C (2022) *In situ* identification of environmental microorganisms with Raman spectroscopy. Environ Sci Ecotechnol 11:100187. 10.1016/j.ese.2022.100187 36158754 10.1016/j.ese.2022.100187 PMC9488013

[CR21] Dalzell DJ, Alte S, Aspichueta E, de la Sota A, Etxebarria J, Gutierrez M, Hoffmann CC, Sales D, Obst U, Christofi N (2002) A comparison of five rapid direct toxicity assessment methods to determine toxicity of pollutants to activated sludge. Chemosphere 47(5):535–545. 10.1016/s0045-6535(01)00331-9 11996129 10.1016/s0045-6535(01)00331-9

[CR22] Davenport R, Curtis-Jackson P, Dalkmann P, Davies J, Fenner K, Hand L, McDonough K, Ott A, Ortega-Calvo JJ, Parsons JR, Schaffer A, Sweetlove C, Trapp S, Wang N, Redman A (2022) Scientific concepts and methods for moving persistence assessments into the 21st century. Integr Environ Assess Manag 18(6):1454–1487. 10.1002/ieam.4575 34989108 10.1002/ieam.4575 PMC9790601

[CR23] Devillers J, Meunier T, Chambon P (1985) Usefulness of the dosage- effect-time relation in ecotoxicology for determination of different chemical classes of toxicants. (Interet de la Relation Dose-Effet-Temps en Ecotoxicologie pour la Determination des Differentes Classes Chimiques de Toxiques). Tech Sci Mun 80:329–334

[CR24] Dib O, Assaf A, Grangé E, Morin J, Cordella C, Thouand G (2023) Automatic recognition of food bacteria using Raman spectroscopy and chemometrics: a comparative study of multivariate models. Vib Spectrosc 126:103535. 10.1016/j.vibspec.2023.103535

[CR25] Dijk JA, Stams AJ, Schraa G, Ballerstedt H, de Bont JA, Gerritse J (2003) Anaerobic oxidation of 2-chloroethanol under denitrifying conditions by *Pseudomonas stutzeri* strain JJ. Appl Microbiol Biotechnol 63(1):68–74. 10.1007/s00253-003-1346-z 12774178 10.1007/s00253-003-1346-z

[CR26] Dimitrov SD, Georgieva DG, Pavlov TS, Karakolev YH, Karamertzanis PG, Rasenberg M, Mekenyan OG (2015) UVCB substances: methodology for structural description and application to fate and hazard assessment. Environ Toxicol Chem 34(11):2450–2462. 10.1002/etc.3100 26053589 10.1002/etc.3100

[CR27] Dutka BJ, Bitton G (1986) Toxicity testing using microorganisms, vol 2. CRC Press, Boca Raton Florida, USA

[CR28] ECHA (2023) Chapter R.7b: endpoint specific guidance. Guidance on information requirements and chemical safety assessment. Version 5.0. December 2023. https://echa.europa.eu/documents/10162/17224/information_requirements_r7b_en.pdf. Accessed 24 Aug 2024

[CR29] Escher BI, Allinson M, Altenburger R, Bain PA, Balaguer P, Busch W, Crago J, Denslow ND, Dopp E, Hilscherova K, Humpage AR, Kumar A, Grimaldi M, Jayasinghe BS, Jarosova B, Jia A, Makarov S, Maruya KA, Medvedev A, Mehinto AC, Mendez JE, Poulsen A, Prochazka E, Richard J, Schifferli A, Schlenk D, Scholz S, Shiraishi F, Snyder S, Su G, Tang JY, van der Burg B, van der Linden SC, Werner I, Westerheide SD, Wong CK, Yang M, Yeung BH, Zhang X, Leusch FD (2014) Benchmarking organic micropollutants in wastewater, recycled water and drinking water with in vitro bioassays. Environ Sci Technol 48(3):1940–1956. 10.1021/es403899t 24369993 10.1021/es403899t

[CR30] Escher BI, Neale PA, Leusch FDL (2021) Bioanalytical tools in water quality assessment. IWA Publishing, London, UK

[CR31] Farias LR, Panero JDS, Riss JSP, Correa APF, Vital MJS, Panero FDS (2023) Rapid and green classification method of bacteria using machine learning and NIR spectroscopy. Sensors (Basel) 23(17). 10.3390/s23177336 10.3390/s23177336 PMC10490430 37687792

[CR32] Farré M, Barcelo D (2003) Toxicity testing of wastewater and sewage sludge by biosensors, bioassays and chemical analysis. Trends Anal Chem 22(5):299–310. 10.1016/S0165-9936(03)00504-1

[CR33] Forney LJ, Liu WT, Guckert JB, Kumagai Y, Namkung E, Nishihara T, RJ L (2001) Structure of microbial communities in activated sludge: potential implications for assessing the biodegradability of chemicals. Ecotoxicol Environ Saf 49:40–53. 10.1006/eesa.2001.2034 10.1006/eesa.2001.2034 11386714

[CR34] Gartiser S, Brunswik-Titze A, Flach F, Junker T, Sättler DJJ (2022) Enhanced ready biodegradability screening tests for the evaluation of potential PBT substances. Sci Total Environ 833:155134. 10.1016/j.scitotenv.2022.155134 35405244 10.1016/j.scitotenv.2022.155134

[CR35] Gartiser S, Brunswik-Titze A, Flach FTJ (2023) Further development of screening tests for the evaluation of potential PBT substances. Final report FKZ 3718 65 410 0 on behalf of the German Environment Agency. UBA Texte 10/2023. https://www.umweltbundesamt.de/sites/default/files/medien/479/publikationen/texte_10-2023_further_development_of_screening_tests_for_the_evaluation_of_potential_pbt_substances.pdf. Accessed 24 Aug 2024

[CR36] Gartiser S, Hafner C, Oeking S, Paschke A (2009) Results of a “whole effluent assessment” study from different industrial sectors in Germany according to OSPAR’s WEA strategy. J Environ Monit 11(2):359–369. 10.1039/B805746J 10.1039/b805746j 19212594

[CR37] Gartiser S, Heisterkamp I, Schoknecht U, Bandow N, Burkhardt NM, Ratte M, Ilvonen O (2017) Recommendation for a test battery for the ecotoxicological evaluation of the environmental safety of construction products. Chemosphere 171:580–587. 10.1016/j.chemosphere.2016.12.115 10.1016/j.chemosphere.2016.12.115 28040614

[CR38] Gatidou G, Chatzopoulos P, Chhetri RA, Kokkoli A, Giannakopoulos A, Andersen HR, Stasiankis AS (2021) Ecotoxicity and biodegradation of the bacteriostatic 3,3',4',5'-tetrachlorosalicylanilide (TSCA) compared to the structurally similar bactericide triclosan. Sci Total Environ 769:144960 10.1016/j.scitotenv.2021.144960 33477039

[CR39] Gendig C, Domogala G, Agnoli F, Pagga U, Strotmann UJ (2003) Evaluation and further development of the activated sludge respiration inhibition test. Chemosphere 52(1):143–149. 10.1016/S0045-6535(03)00111-5 12729697 10.1016/S0045-6535(03)00111-5

[CR40] Goodhead AK, Head IM, Snape JR, Davenport RJ (2014) Standard inocula preparations reduce the bacterial diversity and reliability of regulatory biodegradation tests. Environ Sci Pollut Res 21(16):9511–9521. 10.1007/s11356-013-2064-4 10.1007/s11356-013-2064-4 PMC4133024 24043502

[CR41] Guo G, Huss M, Tong GQ, Wang C, Li Sun L, Clarke ND, Robson P (2010) Resolution of cell fate decisions revealed by single-cell gene expression analysis from zygote to blastocyst. Dev Cell 18(4):675–685. 10.1016/j.devcel.2010.02.012 20412781 10.1016/j.devcel.2010.02.012

[CR42] Gutiérrez M, Etxebarria J, de las Fuentes L (2002) Evaluation of wastewater toxicity: comparative study between Microtox® and activated sludge oxygen uptake inhibition. Water Res 36(4):919–924. 10.1016/S0043-1354(01)00299-8 10.1016/s0043-1354(01)00299-8 11848362

[CR43] Heipieper HJ, Martínez PM (2018) Toxicity of hydrocarbons to microorganisms. In: T K (ed) cellular ecophysiology of microbe: hydrocarbon and lipid interactions. vol 2. Springer International Publishing, Cham, pp 335–344

[CR44] Heipieper HJ, Neumann G, Cornelissen S, Meinhardt F (2007) Solvent-tolerant bacteria for biotransformations in two-phase fermentation systems. Appl Microbiol Biotechnol 74:961–973. 10.1007/s00253-006-0833-4 17262209 10.1007/s00253-006-0833-4

[CR45] Heipieper HJ, Weber FJ, Sikkema J, Keweloh H, Jam DB (1994) Mechanisms behind resistance of whole cells to toxic organic solvents. Trends Biotechnol 12:409–415. 10.1016/0167-7799(94)90029-9

[CR46] Hernando MD, Ejerhoon M, Fernández-Alba ARYC (2003) Combined toxicity effects of MTBE and pesticides measured with *Vibrio fischeri* and *Daphnia magna* bioassays. Water Res 37(17):4091–4098. 10.1016/S0043-1354(03)00348-8 12946890 10.1016/S0043-1354(03)00348-8

[CR47] Hernando MD, Fernandez-Alba AR, Tauler R, Barcelo D (2005) Toxicity assays applied to wastewater treatment. Talanta 65(358-366). 10.1016/j.talanta.2004.07.012 10.1016/j.talanta.2004.07.012 18969807

[CR48] ISO 7827 (2010) Water quality – evaluation of the “ready”, “ultimate” aerobic biodegradability of organic compounds in an aqueous medium – method by analysis of dissolved organic carbon (DOC)

[CR49] ISO 8192 (2007) Water quality – test for inhibition of oxygen consumption by activated sludge for carbonaceous and ammonium oxidation

[CR50] ISO 8692 (2012) Water quality – fresh water algal growth inhibition test with unicellular green algae

[CR51] ISO 9509 (2006) Water quality – toxicity test for assessing the inhibition of nitrification of activated sludge microorganism

[CR52] ISO 10634 (2018) Water quality – preparation and treatment of poorly water-soluble organic compounds for the subsequent evaluation of their biodegradability in an aqueous medium

[CR53] ISO 10712 (1995) Water quality – *Pseudomonas putida* growth inhibition test (*Pseudomonas* cell multiplication inhibition test)

[CR54] ISO 11348 (2008) Water quality – determination of the inhibitory effect of water samples on the light emission of *Vibrio fischeri* (Luminescent bacteria test) – Part 1: method using freshly prepared bacteria – Part 2: method using liquid-dried bacteria – Part 3: method using freeze-dried bacteria

[CR55] ISO 13461 (2003) Water quality – determination of inhibition of gas production of anaerobic bacteria

[CR56] ISO 15522 (1999) Water quality – determination of the inhibitory effect of water constituents on the growth of activated sludge microorganisms

[CR57] ISO 15685 (2012) Soil quality – determination of potential nitrification and inhibition of nitrification – rapid test by ammonium oxidation

[CR58] ISO 18187 (2023) Soil quality – contact test for solid samples using the dehydrogenase activity of *Arthrobacter globiformis*

[CR59] ISO 21338 (2010) Water quality – kinetic determination of the inhibitory effects of sediment, other solids and coloured samples on the light emission of *Vibrio fischeri* (kinetic luminescent bacteria test)

[CR60] ISO 23265 (2023) Soil quality – test for estimating organic matter decomposition in contaminated soil

[CR61] Janssen DB, Scheper A, Witholt B (1984) Biodegradation of 2-chloroethanol and 1,2-dichloroethane by pure bacterial cultures. JIMB 20:169–178

[CR62] Janssen DB, van der Ploeg JR, Pries F (1995) Genetic adaptation of bacteria to halogenated aliphatic compounds. Environ Health Perspect 103 Suppl 5(Suppl 5): 29–32. 10.1289/ehp.95103s429 10.1289/ehp.95103s429 PMC1519299 8565904

[CR63] Järvan M, Edesi L, Adamson A, Vosa T (2014) Soil microbial communities and dehydrogenase activity depending on farming systems. Plant Soil Environ 60(10):459–463. 10.17221/410/2014-PSE

[CR64] Kassem A, Abbas L, Coutinho O, Opara S, Najaf H, Kasperek D, Pokhrel K, Li X, Tiquia-Arashiro S (2023) Applications of Fourier Transform-Infrared spectroscopy in microbial cell biology and environmental microbiology: advances, challenges, and future perspectives. Front Microbiol 14:1304081. 10.3389/fmicb.2023.1304081 38075889 10.3389/fmicb.2023.1304081 PMC10703385

[CR65] Kishino T, Kobayshi K (1996) Studies on the mechanism of toxicity of chlorophenols found in fish through quantitative structure-activity relationships. Water Res 30(2):393–399. 10.1016/0043-1354(95)00152-2

[CR66] Knippschild M, Rehm HJ (1995) Degradation of 2-chloroethanol by free and immobilized *Pseudomonas putida* US2. Appl Microbiol Biotechnol 44:253–258. 10.1007/bf00164511 10.1007/BF00170636 7766127

[CR67] Kowalczyk A, Martin TM, Price OR, Snape JR, van Egmond RA, Finnegan CJ, Schäfer H, Davenport RJ, Bending GD (2015) Refinement of biodegradation test methodologies and the pro- posed utility of new microbial ecology systems. Ecotoxicol Envrion Saf 111:9–22. 10.1016/j.ecoenv.2014.09.021 10.1016/j.ecoenv.2014.09.021 25450910

[CR68] Koziollek P, Knackmuss HJ, Taeger K, Pagga U (1996) A dynamic river model for biodegradability studies. Biodegradation 7:109–120 10.1007/BF00114623 8882804

[CR69] Kutsarova SS, Yordanova DG, Karakolev YH, Stoeva S, Comber M, Hughes CB, Vaiopoulou E, Dimitrov SD, Mekenyan OG (2019) UVCB substances II: development of an endpoint-nonspecific procedure for selection of computationally generated representative constituents. Environ Toxicol Chem 38(3):682–694. 10.1002/etc.4358 30638278 10.1002/etc.4358

[CR70] Lai AL, Clark AM, Escher BI, Ferenadez M, McEwen LR, Tian Z, Wang Z, Schymanksi EL (2022) The next frontier of environmental unknowns: substances of unknown or variable composition, complex reaction products, or biological materials (UVCBs). Environ Sci Technol 56:7448–7466. 10.1021/acs.est.2c00321 10.1021/acs.est.2c00321 PMC9228065 35533312

[CR71] Lappalainen J, Juvonen R, Nurmi J, Karp M (2001) Automated color correction method for Vibrio fischeri toxicity test. Comparison of standard and kinetic assays. Chemosphere 45(4–5):635–41. 10.1016/s0045-6535(00)00579-8 11680759 10.1016/s0045-6535(00)00579-8

[CR72] Lappalainen J, Juvonen R, Vaajasaari K, Karp M (1999) A new flash method for measuring the toxicity of solid and colored samples. Chemosphere 38(5):1069–1073. 10.1016/S0045-6535(98)00352-X

[CR73] Leusch FDL, de Jager C, Levi Y, Lim R, Puijker L, Sacher F, Tremblay LA, Wilson VS, Chapman HF (2010) Comparison of five in vitro bioassays to measure estrogenic activity in environmental waters. Environ Sci Technol 44:3853–3860. 10.1021/ES903899D/SUPPL_FILE/ES903899D_SI_001.PDF 20423077 10.1021/es903899d

[CR74] Leusch FDL, Khan SJ, Gagnon MM, Quayle P, Trinh T, Coleman H, Rawson C, Chapman HF, Blair P, Nice H, Reitsema T (2014) Assessment of wastewater and recycled water quality: a comparison of lines of evidence from *in vitro, in vivo* and chemical analyses. Water Res 50:420–431. 10.1016/J.WATRES.2013.10.056 24210511 10.1016/j.watres.2013.10.056

[CR75] Liu D (1981) A rapid biochemical test for measuring chemical toxicity. Bull Environm Contam Toxicol 26:145–149. 10.1007/bf01622068 10.1007/BF01622068 7248535

[CR76] Liu D (1983) Resazurin reduction method for activated sludge process control. Environ Sci Technol 17:407–411. 10.1021/es00113a009 22239191 10.1021/es00113a009

[CR77] Madoni P (2011) Protozoa in wastewater treatment processes: a minireview. Ital J Zool 78(1):3–11. 10.1080/11250000903373797

[CR78] Maurines-Carboneill C, Pernelle JJ, Morin L, Sachon G, Leblon G (1998) Relevance of the INT test response as an indicator of ETS activity in monitoring heterotrophic aerobic bacterial populations in activated sludges. Water Res 32(4):1213–1221. 10.1016/S0043-1354(97)00329-1

[CR79] Maza-Marquez P, Vilchez-Vargas R, Gonzalez-Martinez A, Gonzalez-Lopez J, Rodelas B (2018) Assessing the abundance of fungal populations in a full-scale membrane bioreactor (MBR) treating urban wastewater by using quantitative PCR (qPCR). J Environ Manage 223:1–8. 10.1016/j.jenvman.2018.05.093 29883777 10.1016/j.jenvman.2018.05.093

[CR80] McCluskey C, Quinn J, McGrath J (2005) An evaluation of three new-generation tetrazolium salts for the measurement of respiratory activity in activated sludge microorganisms. Microb Ecol 49:379–387 16003480 10.1007/s00248-004-0012-z

[CR81] Mendonca E, Picado A, Paixao SM, Silva L, Cunha MA, Leitao S, Moura I, Cortez C, Brito F (2009) Ecotoxicity tests in the environmental analysis of wastewater treatment plants: case study in Portugal. J Hazard Mater 163(2–3):665–670. 10.1016/j.jhazmat.2008.07.012 18691813 10.1016/j.jhazmat.2008.07.012

[CR82] Menz J, Schneider M, Kummerer K (2013) Toxicity testing with luminescent bacteria-characterization of an automated method for the combined assessment of acute and chronic effects. Chemosphere 93(6):990–996. 10.1016/j.chemosphere.2013.05.067 23806483 10.1016/j.chemosphere.2013.05.067

[CR83] Mizukami-Murata S, Takanashi H, Sawai A, Suzuki Y, Tsushima I, Yamashita H, Goto Y, Toda M (2023) Characteristics of compounds with strong or weak nitrification inhibition in sewage. Environ Monit Assess 195(12):1437. 10.1007/s10661-023-12074-z 37940732 10.1007/s10661-023-12074-z

[CR84] Muñoz-Palazon B, Pesciaroli C, Rodriguez-Sanchez A, Gonzalez-Lopez J, Gonzalez-Martinez A (2018) Pollutants degradation performance and microbial community structure of aerobic granular sludge systems using inoculums adapted at mild and low temperature. Chemosphere 204:431–441. 10.1016/j.chemosphere.2018.04.062 29677650 10.1016/j.chemosphere.2018.04.062

[CR85] Nabeoka R, Kameya T, Yoshida T, Kayashima T (2020) Effects of adsorbent carriers in modified ready biodegradability tests of quaternary ammonium salts. J Environ Sci Health 55(11):1294–1303. 10.1080/10934529.2020.1789410 10.1080/10934529.2020.1789410 32657211

[CR86] Nishigaya Y, Fujimoto Z, Yamazaki T (2016) Optimized inhibition assays reveal different inhibitory responses of hydroxylamine oxidoreductases from beta- and gamma-proteobacterial ammonium-oxidizing bacteria. Biochem Biophys Res Commun 476(3):127–133. 10.1016/j.bbrc.2016.05.041 27173879 10.1016/j.bbrc.2016.05.041

[CR87] O’Malley L (2006) Evaluation and modification of the OECD 301 F respirometry biodegradation test method with regard to test substance concentration and inoculum. Water Air Soil Pollut 177:251–265. 10.1007/s11270-006-9163-5

[CR88] OECD 201 (2006) Guidelines for the testing of chemicals - freshwater alga and cyanobacteria, growth inhibition test

[CR89] OECD 209 (2010) Activated sludge, respiration inhibition test (carbon and ammonium oxidation)

[CR90] OECD 216 (2000) Soil microorganisms: nitrogen transformation test

[CR91] OECD 217 (2000) Soil microorganisms: carbon transformation test

[CR92] OECD 224 (2007) Guidelines for the testing of chemicals - determination of the inhibition of the activity of anaerobic bacteria – reduction of gas production from anaerobically digesting (sewage) sludge

[CR93] OECD 301 (1992) Guidelines for the testing of chemicals - ready biodegradability

[CR94] OECD 301 A (1992) Ready biodegradability - DOC die-away test

[CR95] OECD 301 B (1992) Ready biodegradability - CO2 evolution test (modified sturm test)

[CR96] OECD 301 C (1992) Ready biodegradability - MITI (I) test

[CR97] OECD 301 D (1992) Ready biodegradability - closed bottle test

[CR98] OECD 301 E (1992) Ready biodegradability - modified OECD screening test

[CR99] OECD 301 F (1992) Ready biodegradability - manometric respirometry test

[CR100] OECD 302 A (1981) Inherent biodegradability - modified SCAS test

[CR101] OECD 302 B (1992) Inherent biodegradability - Zahn-Wellens / EMPA test

[CR102] OECD 302 C (1981) Inherent biodegradability - modified MITI (II) test

[CR103] OECD 304 A (1981) Inherent biodegradability in soil

[CR104] Oliveira CA, Araujo CVM, Nascimento RB, Strotmann UJ, da Silva EM (2007) Utilization of respirometry to assess organic matter reduction of effluents from the Camacari industrial complex (BA, Bahia). Braz Arch Biol Technol 50(2):311–319. 10.1590/S1516-89132007000200016

[CR105] Overmeyer C, Rehm HJ (1995) Biodegradation of 2-chloroethanol by freely suspended and adsorbed immobilized *Pseudomonas putida* US2 in soil. Appl Microbiol Biotechnol 43(1):143–149. 10.1007/BF00170636 7766127 10.1007/BF00170636

[CR106] Pagga U (1981) Der Kurzzeitatmungstest - eine einfache Methode zur Bestimmung der Atmungsaktivität von Belebtschlamm. Vom Wasser 57:263–275

[CR107] Pagga U (1983) Die Bedeutung von Biotests für die Toxizitätsbestimmung von Abwasser. Wasserwirtschaft 73:65–70

[CR108] Pagga U (1985) Stoffprüfungen in einem Kläranlagenmodell - Abbaubarkeits- und Toxizitätstests im BASF-Toximeter. Z Wasser-Abwasser-Forsch 18:222–232

[CR109] Pagga U (1986) Biologische Abwasserüberwachung in einem Großbetrieb der Chemischen Industrie. Vom Wasser 67:83–98

[CR110] Pagga U (1997) Testing biodegradability with standardized method. Chemosphere 35:2953–2972. 10.1016/S0045-6535(97)00262-2 9415981 10.1016/s0045-6535(97)00262-2

[CR111] Pagga U, Bachner J, Strotmann U (2006) Inhibition of nitrification in laboratory tests and model wastewater treatment plants. Chemosphere 65(1):1–8. 10.1016/j.chemosphere.2006.03.021 16635505 10.1016/j.chemosphere.2006.03.021

[CR112] Pagga U, Günthner W (1981) The BASF toximeter - a helpful instrument to control and monitor biological waste water treatment plants. Wat Sci Tech 13:233–238

[CR113] Pagga U, Günthner W, Homans WJ (1986) Überwachung biologischer Kläranlagen vol Band 92, München

[CR114] Pagga U, Strotmann U (1999) Bakterientoxizität – standardisierte Testmethoden und Erfahrungen. gwf Wasser Abwasser 140(12):827–835

[CR115] Painter H (1995) Detailed review paper on biodegradability testing OECD Series on the test guidelines programme Environment monograph No 98 OECD Paris. https://one.oecd.org/document/ocde/gd(95)43/en/pdf. Accessed 24 Aug 2024

[CR116] Paixao SM, Silva L, Fernandes A, O’Rourke K, Mendonca E, Picado A (2008) Performance of a miniaturized algal bioassay in phytotoxicity screening. Ecotoxicology 17(3):165–171. 10.1007/s10646-007-0179-4 17978872 10.1007/s10646-007-0179-4

[CR117] Pan Y, Yu SS, Xiao ZC, Min Y, Tian T, Zheng YM, Zhao QB, Yuan ZH, Yu HQ (2023) Re-evaluation and modification of dehydrogenase activity tests in assessing microbial activity for wastewater treatment plant operation. Water Res 246:120737. 10.1016/j.watres.2023.120737 37857011 10.1016/j.watres.2023.120737

[CR118] Pedrazzani R, Ceretti E, Zerbini I, Casale R, Gozio E, Bertanza G, Gelatti U, Donato F, Feretti D (2012) Biodegradability, toxicity and mutagenicity of detergents: integrated experimental evaluations. Ecotoxicol Env Saf 84:274–281. 10.1016/j.econv2012.07.23 10.1016/j.ecoenv.2012.07.023 22898309

[CR119] Poursat BAJ, van Spanning RJM, Braster M, Helmus R, de Voogt P, Parsons JR (2020) Long-term exposure of activated sludge in chemostats leads to changes in microbial communities composition and enhanced biodegradation of 4-chloroaniline and N-methylpiperazine. Chemosphere 242:125102. 10.1016/j.chemosphere.2019.125102 31669985 10.1016/j.chemosphere.2019.125102

[CR120] Poursat BAJ, van Spanning RJM, de Voogt P, Parsons JR (2019) Implications of microbial adaptation for the assessment of environmental persistence of chemicals. Crit Rev Environ Sci Technol 1547–6537. 10.1080/10643389.2019.1607687

[CR121] Prosser CM, Davis CW, Bragin GE, Camenzuli L (2023) Using weight of evidence to assess degradation potential of UVCB hydrocarbon solvents. Integr Environ Assess Manag 19(4):1120–1130. 10.1002/ieam.4731 36600450 10.1002/ieam.4731

[CR122] Prosser JI, Bohannan BJ, Curtis TP, Ellis RJ, Firestone MK, Freckleton RP, Green JL, Green LE, Killham K, Lennon JJ, Osborn AM, Solan M, van der Gast CJ, Young JP (2007) The role of ecological theory in microbial ecology. Nat Rev Microbiol 5(5):384–392. 10.1038/nrmicro1643 17435792 10.1038/nrmicro1643

[CR123] Ren S, Frymier PD (2003) Use of multidimensional scaling in the selection of wastewater toxicity test battery components. Water Res 37:1655–1661. 10.1016/S0043-1354(02)00518-3 12600394 10.1016/S0043-1354(02)00518-3

[CR124] Reuschenbach P, Pagga U, Strotmann U (2003) A critical comparison of respirometric biodegradation tests based on OECD 301 and related test methods. Water Res 37(7):1571–1582. 10.1016/S0043-1354(02)00528-6 12600385 10.1016/S0043-1354(02)00528-6

[CR125] Reynolds L, Blok J, de Morsier A, Gerike P, Wellens H, Bontinck WJ (1987) Evaluation of the toxicity of substances to be assessed for biodegradability. Chemosphere 16:2259–2277. 10.1016/0045-6535(87)90284-0

[CR126] Riedel R, Krahl K, Buder K, Bollmann J, Braun B, Martienssen M (2023) Novel standard biodegradation test for synthetic phosphonates. J Microbiol Methods 212:106793. 10.1016/j.mimet.2023.106793 37543110 10.1016/j.mimet.2023.106793

[CR127] Rodriguez L, Zhang Z, Wang D (2023) Recent advances of Raman spectroscopy for the analysis of bacteria. Anal Sci Adv 4(3–4):81–95. 10.1002/ansa.202200066 38715923 10.1002/ansa.202200066 PMC10989577

[CR128] Sagi G, Bezsenyi A, Kovacs K, Klatyk S, Darvas B, Szekacs, A, Mohasci-Farkas C, Takacs E, Wojnarovits L (2018) Radiolysis of sulfonamide antibiotics in aqueous solution: degradation efficiency and assessment of antibactrial acitivity, toxicity and biodegradability of products. Sci Total Environ 622–623:1009–1015 10.1016/j.scitotenv.2017.12.065 29890571

[CR129] Saunders AM, Albertsen M, Vollertsen J, Nielsen PH (2016) The activated sludge ecosystem contains a core community of abundant organisms. ISME J 10:11–20. 10.1038/ismej.2015.117 26262816 10.1038/ismej.2015.117 PMC4681854

[CR130] Scheringer M, Strempel S, Hukari S, Ng CA, Blepp M, Hungerbühler K (2012) How many persistent organic pollutants should we expect? Atmos Pollut Res 3:383–391. 10.5094/APR.2012.044

[CR131] Shigeoka T, Yamagata T, Minoda T, Yamauchi F (1988) Acute toxicity and hatching inhibition of chlorophenols to Japanese medaka, *Oryzias latipes* and structure-activity relationships. Eisei Kagaku 34(4):343–349. 10.1248/jhs1956.34.343

[CR132] Shin NR, Whon TW, Bae JW (2015) Proteobacteria: microbial signature of dysbiosis in gut microbiota. Trends Biotechnol 33(9):496–503. 10.1016/j.tibtech.2015.06.011 26210164 10.1016/j.tibtech.2015.06.011

[CR133] Sigma-Aldrich (2024) Safety data sheet 3.5-Dichlorophenol. Version 6.5. Revision Date 2.06.2023. (22.12.2023)

[CR134] Sikkema JAN, de Bont JA, Poolman B (1995) Mechanisms of membrane toxicity of hydrocarbons. Microbiol Rev 59:201–222. 10.1128/mr.59.2.201-222.1995 7603409 10.1128/mr.59.2.201-222.1995 PMC239360

[CR135] Smital T, Terzic S, Zaja R, Senta I, Pivcevic B, Popovic M, Mikac I, Tollefsen KE, Thomas KV, Ahel M (2011) Assessment of toxicological profiles of the municipal wastewater effluents using chemical analyses and bioassays. Ecotoxicol Environ Saf 74(4):844–851. 10.1016/j.ecoenv.2010.11.010 21159381 10.1016/j.ecoenv.2010.11.010

[CR136] Smith S, Furay VJ, Layiwola PJ, Menezesfilho JA (1994) Evaluation of the toxicity and quantitative structure-activity-relationships (QSAR) of chlorophenols to the copepodid stage of a marine copepod (*tisbe-battagliai*) and 2 species of benthic flatfish, the flounder (P*latichthys flesus*) and sole (*Solea solea*). Chemosphere 28(4):825–836. 10.1016/0045-6535(94)90234-8

[CR137] Stalter D, Magdeburg A, Wagner M, Oehlmann J (2011) Ozonation and activated carbon treatment of sewage effluents: removal of endocrine activity and cytotoxicity. Water Res 45:1015–1024. 10.1016/J.WATRES.2010.10.008 21074820 10.1016/j.watres.2010.10.008

[CR138] Stolte S, Steudte, S, Areitioaurtena O, Pagano F, Thöming J, Stepnowski P, Igartua A (2012) Ionic liquids as lubricants or lubrication additives: an ecotoxicity and biodegradability assessment. Chemosphere 89(9):1135–1141. 10.1016/j.chemosphere.201205.102 10.1016/j.chemosphere.2012.05.102 22749125

[CR139] Strempel S, Scheringer M, Ng CA, Hungerbühler K (2012) Screening for PBT chemicals among the “existing” and “new” chemicals of the EU. Environ Sci Technol 46:5680. 10.1021/es3002713 22494215 10.1021/es3002713

[CR140] Strotmann U, Butz B, Bias WR (1993a) The dehydrogenase assay with resazurin: practical performance as a monitoring system and Ph-dependent toxicity of phenolic compounds. Ecotoxicol Environ Saf 25(1):79–89. 10.1006/eesa.1993.1009 7682921 10.1006/eesa.1993.1009

[CR141] Strotmann UJ, Eglsäer H (1995) The toxicity of substituted phenols in the nitrification inhibition test and luminescent bacteria test. Ecotoxicol Env Saf 30(3):269–273 10.1006/eesa.1995.1030 7541340

[CR142] Strotmann U, Eglsäer H, Pagga U (1994) Development and evaluation of a growth inhibition test with sewage bacteria for assessing bacterial toxicity of chemical compounds. Chemosphere 28(4):755–766. 10.1016/0045-6535(94)90229-1

[CR143] Strotmann U, Eismann F, Hauth B, Bias WR (1993b) An integrated test strategy for the assessment of anaerobic biodegradability of wastewaters. Chemosphere 26(12):2241–2254. 10.1016/0045-6535(93)90350-E

[CR144] Strotmann U, Pagga U (1996) A growth inhibition test with sewage bacteria–results of an international ring test 1995. Chemosphere 32(5):921–933. 10.1016/0045-6535(95)00357-6 8867142 10.1016/0045-6535(95)00357-6

[CR145] Strotmann U, Pastor Flores D, Konrad O, Gendig C (2020) Bacterial toxicity testing: modification and evaluation of the luminescent bacteria test and the respiration inhibition test. Processes 8(11):1349. 10.3390/pr8111349

[CR146] Strotmann U, Pentenga M, Janssen DB (1990) Degradation of 2-chloroethanol by wild type and mutants of *Pseudomonas putida* US2. Arch Microbiol 154(3):294–300. 10.1007/BF00248970

[CR147] Strotmann U, Röschenthaler R (1987) A method for screening bacteria: aerobically degrading chlorinated short-chain hydrocarbons. Curr Microbiol 15:159–163. 10.1007/bf01577266

[CR148] Strotmann U, Thouand G, Pagga U, Gartiser S, Heipieper HJ (2023) Toward the future of OECD/ISO biodegradability testing - new approaches and developments. Appl Microbiol Biotechnol 107(7–8):2073–2095. 10.1007/s00253-023-12406-6 36867202 10.1007/s00253-023-12406-6 PMC10033483

[CR149] Strotmann U, Weberruß U, Bias WR (1993c) Degradation of morpholine in several biodegradation tests and in wastewater treatment plants. Chemosphere 26(9):1729–1742. 10.1016/0045-6535(93)90116-M

[CR150] Strotmann U, Weisbrodt W (1994) Wastewater treatment and integrated environmental protection at the BASF AG in Ludwigshafen, Germany. Wat Sci Tech 29(8):185–192. 10.2166/wst.1994.0407

[CR151] Strotmann U, Zaremba S, Bias WR (1992) Rapid toxicity test systems for the determination of susbstance toxicity to activated sludge. Acta Hydrochim Hydrobiol 20(3). 10.1002/aheh.19920.200302

[CR152] Struijs J, Stoltenkamp-Wouterse MJ, Dekkers ALM (1995) A rationale for the appropriate amount of inoculum in ready biodegradability tests. Biodegradation 6(4):319–327. 10.1007/BF00695262 8580645 10.1007/BF00695262

[CR153] Tchobanoglous G, Burton FL, Eddy M (1991) Wastewater engineering: treatment, disposal and reuse, 3rd edition. Mc Graw Hill New York

[CR154] Thouand G, Block JC (1993) The use of precultured inocula for biodegradability tests. Environ Techn (UK) 14(7):601–614. 10.1080/09593339309385330

[CR155] Thouand G, Durand MJ, Maul A, Gancet CHB (2011) New concept in the evaluation of biodegradation/persistence of chemical substances using a microbial inoculum. Front Microbiol 2:1–6. 10.3389/fmicb.2011.00164 21863143 10.3389/fmicb.2011.00164 PMC3149152

[CR156] Thouand G, Friant P, Bois F, Cartier A, Maul A, Block JC (1995) Bacterial inoculum density and probability of para-nitrophenol biodegradability test response. Ecotoxicol Environ Saf 30(3):274–282. 10.1006/eesa.1995.1031 7541341 10.1006/eesa.1995.1031

[CR157] Timmer N, Gore D, Sanders D, Gouin T, Droge STJ (2019) Sorbent-modified biodegradation studies of the biocidal cationic surfactant cetylpyridinium chloride. Ecotoxicol Environ Saf 182:109417 31302333 10.1016/j.ecoenv.2019.109417

[CR158] UBA (1993) Entwurf zur Bewertung von Bodenbelastungen, Fachgebiet I 3.7. 7 Umweltbundesamt Berlin in: guidance on information requirements and chemical safety assessment Chapter R.10: Characterisation of dose [concentration]-response for environment. https://echa.europa.eu/documents/10162/13632/information_requirements_r10_en.pdf/bb902be7-a503-4ab7-9036-d866b8ddce69. Accessed 24 Aug 2024

[CR159] Välitalo P, Massei R, Heiskanen I, Behnisch P, Brack W, Tindall AJ, du Pasquier D, Küster E, Mikola A, Schulze T, Sillanpää M (2017) Effect-based assessment of toxicity removal during wastewater treatment. Water Res 126:153–163. 10.1016/J.WATRES.2017.09.014 28941401 10.1016/j.watres.2017.09.014

[CR160] van Ginkel CG, Gancet C, Hirschen M, Galobardes M, Lemaire P, Rosenblom J (2008) Improving ready biodegradability testing of fatty amine derivatives. Chemosphere 73(4):506–510. 10.1016/j.chemosphere.2008.06.037 18674795 10.1016/j.chemosphere.2008.06.037

[CR161] Vázquez-Rodríguez GA, Garabétian F, Rols J-L (2007) Inocula from activated sludge for ready biodegradability testing: homogenization by preconditioning. Chemosphere 68(8):1447–1454. 10.1016/j.chemosphere.2007.03.073 17509642 10.1016/j.chemosphere.2007.03.073

[CR162] Wang S, Wang X, Fessler M, Jin B, Su Y, Zhang Y (2022) Insights into the impact of polyethylene microplastics on methane recovery from wastewater via bioelectrochemical anaerobic digestion. Water Res 221:118844. 10.1016/j.watres.2022.118844 35949067 10.1016/j.watres.2022.118844

[CR163] Wang Z, Walker GW, Muir DCG, Nagatani-Yoshida K (2020) Toward a global understanding of chemical pollution: a first comprehensive analysis of national and regional chemical inventories. Environ Sci Technol 54:2575–2584. 10.1021/acs.est.9b06379 31968937 10.1021/acs.est.9b06379

[CR164] Wu L, Ning D, Zhang B, Li Y, Zhang P, Shan X, Zhang Q, Brown MR, Li Z, Van Nostrand JD, Ling F, Xiao N, Zhang Y, Vierheilig J, Wells GF, Yang Y, Deng Y, Tu Q, Wang A, Global Water Microbiome C, Zhang T, He Z, Keller J, Nielsen PH, Alvarez PJJ, Criddle CS, Wagner M, Tiedje JM, He Q, Curtis TP, Stahl DA, Alvarez-Cohen L, Rittmann BE, Wen X, Zhou J (2019) Global diversity and biogeography of bacterial communities in wastewater treatment plants. Nat Microbiol 4(7):1183-1195. 10.1038/s41564-019-0426-5 10.1038/s41564-019-0426-5 31086312

[CR165] Xia S, Duan L, Song Y, Li J, Piceno YM, Andersen GL, Alvarez-Cohen L, Moreno-Andrade I, Huang CL, Hermanowicz SW (2010) Bacterial community structure in geographically distributed biological wastewater treatment reactors. Environ Sci Technol 44(19):7391–7396. 10.1021/es101554m 20812670 10.1021/es101554m

[CR166] Xie J, Hu W, Pei H, Dun M, Qi F (2008) Detection of amount and activity of living algae in fresh water by dehydrogenase activity (DHA). Environ Monit Assess 146:473–478. 10.1007/s10661-008-0250-5 18398692 10.1007/s10661-008-0250-5

[CR167] Yuan Y, Yin Y, Hongbo X, Zhou Y, He X (2019) Comparison of four test methods for toxicity evaluation of typical toxicants in petrochemical wastewater on activated sludge. Sci Total Environ 685:273–279. 10.1016/j.scitotenv.2019.05.389 31176214 10.1016/j.scitotenv.2019.05.389

[CR168] Zhang B, Xu X, Zhu L (2018) Activated sludge bacterial communities of typical wastewater treatment plants: distinct genera identification and metabolic potential differential analysis. AMB Express 8:184. 10.1186/s13568-018-0714-0 30430271 10.1186/s13568-018-0714-0 PMC6236004

[CR169] Zhang K, Liu M, Song X, Wang D (2023) Application of luminescent bacteria bioassay in the detection of pollutants in soil. Sustainability 15:7351. 10.3390/su15097351

[CR170] Zhang T, Shao MF, Ye L (2012) 454 Pyrosequencing reveals bacterial diversity of activated sludge from 14 sewage treatment plants. ISME J 6:1137–1147. 10.1038/ismej.2011.188 22170428 10.1038/ismej.2011.188 PMC3358032

[CR171] Zhang Y, Freedman ZB, Hartemink AE, Whitman T, Huang J (2022) Characterizing soil microbial properties using MIR spectra across 12 ecoclimatic zones (NEON sites). Geoderma 409(115647). 10.1016/j.geoderma.2021.115647

