# Supplementary material for: Microbiological toxicity tests using standardized ISO/OECD methods—current state and outlook
Source: Appl Microbiol Biotechnol. 2024 Aug 31;108:454. doi: 10.1007/s00253-024-13286-0 (PMC11365844; doi:10.1007/s00253-024-13286-0)
Supplement: Supplementary file 1 [file 253_2024_13286_MOESM1_ESM.pdf]

Supplementary Material

Microbiological toxicity tests using standardized ISO/OECD methods – current state and outlook

Uwe Strotmann[1], Marie-José Durand[2], Gerald Thouand[2], Christian Eberlein[3], Hermann J. Heipieper[3]*, Stefan Gartiser[4], Udo Pagga[5]

[1]Westfälische Hochschule, Dept. of Chemistry, Recklinghausen, Germany (uwe.strotmann@w-hs.de), [2]Nantes Université, ONIRIS, CNRS, GEPEA, UMR 6144, 85000 La Roche sur Yon, France, France, (marie-josé.durand-thouand@univ-nantes, gerald.thouand@univ-nantes.fr) [3]Department of Molecular Environmental Biotechnology, Helmholtz Centre for Environmental Research - UFZ, Leipzig, Germany (hermann.heipieper@ufz.de, christian.eberlein@ufz.de)

[4] Hydrotox GmbH, Bötzinger Str. 29, 79111 Freiburg, Germany (gartiser@hydrotox.de)

[5]Rüdigerstr. 49, 67069 Ludwigshafen, Germany (udo.pagga@t-online.de)

*corresponding author: Hermann J. Heipieper, hermann.heipieper@ufz.de

Table S1. EC values in the respiration inhibition test with municipal and industrial sludge using selected dichlorophenols (DCPs) (modified after Strotmann et al., 2020; Strotmann et al., 1994) at different incubation times. All EC values are given in mg L^{-1}).

	2,3-DCP	3,4-DCP	3,5-DCP
Municipal sludge (27 h test)			
EC_{20}	0.4	0.4	0.6
EC_{50}	5.5	3.4	6.4
EC_{80}	52	21.4	27.2
Industrial sludge (30 min test)			
EC_{20}	32	10	6
EC_{50}	120	15	10
EC_{80}	320	38	34

Table S2. EC_{50} values (in mg L^{-1}) of phenolic compounds in the dehydrogenase test at different pH values (modified after Strotmann et al., 1993).

Compound	pH	EC_{50} (mg L^{-1})
Phenol	5.5	>100
	6.0	>100
	6.5	>100
	7.0	>100
	7.5	>100
3-Chlorophenol	5.5	>100
	6.0	>100
	6.5	>100
	7.0	>100
	7.5	>100
2,4-Dichlorophenol	5.5	35
	6.0	70
	6.5	>100
	7.0	>100
	7.5	>100
3,5-Dichlorophenol	5.5	5
	6.0	15
	6.5	33
	7.0	>100
	7.5	>100
2,4-Dinitrophenol	5.5	4
	6.0	10
	6.5	95
	7.0	>100
	7.5	>100

Table S3. Detailed ecotoxicity and biodegradation data from a bibliographic analysis

Reference	OECD Biodegradation tests	Microbial assay: Respiration inhibition OECD 209	Microbial assay: Growth inhibition (*Pseud. putida*, ISO 10712)	Microbial assay: Luminescence inhibition (*Aliivibrio fischeri*, ISO 11348)	Microbial assay: Mutagenicity test (*Salmonella typhimurium*): Ames	Microbial assay: Mutagenicity test (*Salmonella typhimurium*): UMU test	Enzyme: Inhibition (ATP/ AchE)	Eukaryotic cells: Cell viability: Mammalian	Eukaryotic cells: Cell viability: Fish cell biomarkers	Eukaryotic cells: Genotoxic effect: HeLa cell	Photosynthetic species: Growth inhibition: Green microalgae OECD 201	Photosynthetic species: Growth inhibition: *Lemna minor* ISO 20079:2005	Photosynthetic species: Seed germination /root elongation: OECD 208	Photosynthetic species: Seed germination /root elongation: ISO 11269-1,1993	Photosynthetic species: Micronucleus (*Alium cepa*)	Invertebrate species: Acute toxicity *Daphnia magna* OECD 202	Invertebrate species: Chronic toxicity *Daphnia magna* OECD 211	Invertebrate species: Acute Toxicity Earthworm OECD 207	Vertebrate species: Acute toxicity (fresh water fish, OECD 203): *Brachydanio rerio*	Vertebrate species: Acute toxicity (fresh water fish, OECD 203): *Primephales promelas*	Vertebrate species: Acute toxicity (fresh water fish, OECD 203): Other fish	Vertebrate species: Fish eggs OECD 210
Reynolds et al, 1987	301B, 301C, 301E	☒		☒																		
Giolando et al, 1995	301B	☒									☒		☒			☒	☒	☒	☒	☒		
Murin et al, 1997	301C, 301D										☒					☒						
Bowmer at al, 1998	301C, 301D										☒					☒			☒	☒		
Steger-Hartmann et al, 1999	301E		☒	☒							☒					☒	☒		☒		☒	
Straub, 2009	303A, 308, 309										☒						☒					☒
Jurado et al, 2009	301E			☒ ⊠																		
Garcia et al, 2009	310			☒												☒						
Chatzisymeon et al, 2009	302B			☒ ⊠																		
Coelho et al, 2009	302B	☒		☒ ⊠																		
Gartiser et al, 2009	301 A, 302B,			☒ ⊠	☒ ⊠	☒ ⊠					☒ ⊠					☒ ⊠						☒ ⊠
Pessala et al, 2009	310						☒		☒ ⊠													
Weltman et al, 2011	301B	☒									☒						☒			☒		
Berghein et al, 2012	301D, 301F, 302B		☒																			
Pedrazzani et al, 2012	301 F			☒	☒										☒	☒						
Pietka -Ottlik et al, 2012	301F			☒																		
Russo et al, 2012	304 modified			☒ ⊠									☒ ⊠	☒ ⊠								
Stolte et al, 2012	301B,301F			☒			☒	☒			☒						☒					
Peric et al, 2013	301F						☒	☒			☒	☒										
Sanchi et al, 2013	301F			☒																		
Bergheim et al, 2015	301D, 301F, 302B		☒	☒				☒		☒												
Herrmann et al, 2015	301D			☒	☒																	
Radošević et al, 2015	301D			☒				☒					☒									
Campisi et al, 2016	301F										☒					☒						
Gartiser et al, 2017	301 F			☒		☒					☒											☒
Menz et al, 2017	301 F	☒	☒	☒																		
Wilde et al, 2017	301 D,301F			☒																		
Sagi et al, 2018	302 F	☒		☒							☒					☒						
Mustahil et al, 2019	301D		☒																			
Garcia et al, 2019	310										☒					☒						
Busto et al, 2020	311			☒ ⊠							☒ ⊠		☒ ⊠									
Gomez-Herrero et al, 2020	302B	☒		☒																		
Mena et al, 2020	302B	☒		☒																		
Gatidou et al, 2021	301E	☒		☒								☒				☒						
Busto et al, 2022	311			⊠							⊠		⊠									
Guerreiro et al, 2022	301B	☒									☒					☒						
Mengata et al, 2022	301B																	☒			☒	
Sousa et al, 2023	301															☒						
Puhlmann et al, 2024	301F			☒																		

☒ : tests carried out on compound before biodegradation test
⊠: tests carried out on compound after biodegradation test
The studies are listed in chronological order.

Table S4. The BASF wastewater treatment plant. Operational parameters of the aeration units and performance of the plant (average data, modified after Strotmann and Weisbrodt, 1994)

**Data about the aeration basins**			
Parameter	Content	Unit	
Volume	300,000	m^3	
Hydraulic residence time	12	h	
Activated sludge content (MLSS)	4 - 5	g L^{-1}	
Volumetric loading rate	1.8	kg COD m^{-3} d^{-1}	
Sludge loading rate	0.4	kg COD kg MLSS-^{1} d^{-1}	
Temperature (°C)	20 -30		
pH in the aeration basins	6.9 – 7.2		
**Efficiency**			
Parameter	Influent	Effluent	Removal (%)
COD (mg L^{-1})	1000	120	88
TOC (mg L^{-1})	333	40	88
BOD_5 (mg L^{-1})	500	10	98
Nitrate (mg L^{-1})	150	1.5	99

Fig. S1. Strategy of the bibliographic analysis concerning ecotoxicity and biodegradation

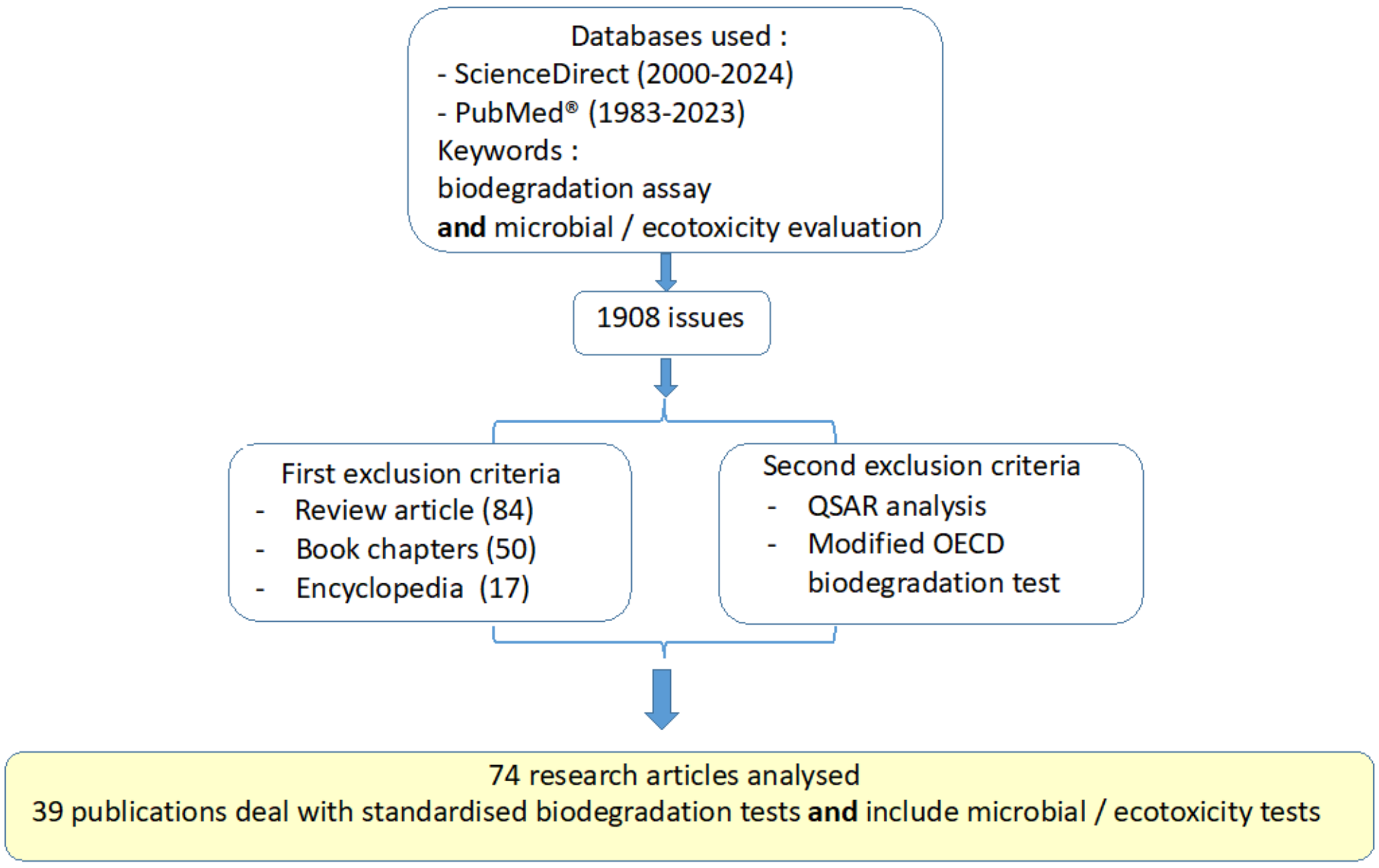

References Supplementary Material

Bergheim M, Giere R, Kümmerer K (2012) Biodegradability and ecotoxicitiy of tramadol, ranitidine, and their photoderivatives in the aquatic environment. Environ Sci Pollut Res 19(1):72-85 doi:10.1007/s11356-011-0536-y

Bergheim M, Gminski, R, Spangenberg, B, Debiak, M, Bürkle, A, Mersch-Sundermann, V, Kümmerer, K, Gieré, R (2015) Antibiotics and Sweeteners in the Aquatic Environment: Biodegradability, Formation of Phototransformation Products, and in Vitro Toxicity. Environ Sci Pollut Res 22:18017-18030 doi: https://doi.org/10.1007/s11356-015-4831-x

Bowmer CT, Hooftman RN, Hanstveit AO, Venderbosch PW, van der Hoeven N (1998) The ecotoxicity and the biodegradability of lactic acid, alkyl lactate esters and lactate salts. Chemosphere 37(7):1317-33 doi:10.1016/s0045-6535(98)00116-7

Busto RV, Hunter C, Roberts J, Escudero A, Helwig K, Pahl O, Coelho LHG (2022) Anaerobic biodegradation of dipyrone: Determination of transformation products by LC-HRMS and ecotoxicity assessment using target organisms and QSAR model. J Environ Chem Eng 10(3) doi:ARTN 10774010.1016/j.jece.2022.107740

Busto RV, Roberts J, Hunter C, Escudero A, Helwig K, Coelho LHG (2020) Mechanistic and ecotoxicological studies of amoxicillin removal through anaerobic degradation systems. Ecotoxicol Environ Saf 192:110207 doi:10.1016/j.ecoenv.2020.110207

Campisi T, Samori C, Torri C, Barbera G, Foschini A, Kiwan A, Galletti P, Tagliavini E, Pasteris A (2016) Chemical and ecotoxicological properties of three bio-oils from pyrolysis of biomasses. Ecotoxicol Environ Saf 132:87-93 doi:10.1016/j.ecoenv.2016.05.027

Chatzisymeon E, Xekoukoulotakis NP, Diamadopoulos E, Katsaounis A, Mantzavinos D (2009) Boron-doped diamond anodic treatment of olive mill wastewaters: statistical analysis, kinetic modeling and biodegradability. Water Res 43(16):3999-4009 doi:10.1016/j.watres.2009.04.007

Coelho AD, Sans C, Aguera A, Gomez MJ, Esplugas S, Dezotti M (2009) Effects of ozone pre-treatment on diclofenac: intermediates, biodegradability and toxicity assessment. Sci Total Environ 407(11):3572-8 doi:10.1016/j.scitotenv.2009.01.013

de Sousa TAT, Dantas ERB, Lopes WDS, Leite VD, Sousa JT, Lopes WS (2023) Toxicity assessment of sanitary landfill leachate before and after Fenton treatment process. Sci Total Environ 893:164870 doi:10.1016/j.scitotenv.2023.164870

Garcia MT, Ribosa I, Kowalczyk I, Pakiet M, Brycki B (2019) Biodegradability and aquatic toxicity of new cleavable betainate cationic oligomeric surfactants. J Hazard Mater 371:108-114 doi:10.1016/j.jhazmat.2019.03.005

Gartiser S, Hafner, C, Oeking, S, Paschke, A (2009) Results of a "Whole Effluent Assessment" Study from Different Industrial Sectors in Germany According to OSPAR's WEA Strategy. . J Environ Monit 11(2):359-369 doi: https://doi.org/10.1039/B805746J

Gartiser S, Heisterkamp, I, Schoknecht, U, Bandow, N, Burkhardt, NM,Ratte, M, Ilvonen, O (2017) Recommendation for a Test Battery for the Ecotoxicological Evaluation of the Environmental Safety of Construction Products. Chemosphere 171:580-587 doi:https://doi.org/10.1016/j.chemosphere.2016.12.115

Gatidou G, Chatzopoulos, P, Chhetri, RA, Argyro, C, Kokkoli, A, Giannakopoulos, A, Andersen, HR, Stasinakis, AS (2021) Ecotoxicity and biodegradation of the bacteriostatic 3,3′,4′,5-tetrachlorosalicylanilide (TSCA) compared to the structurally similar bactericide triclosan. Sci Total Environ 769:144960 doi:https://doi.org/10.1016/j.scitotenv.2021.144960

Giolando ST, Rapaport RA, Larson RJ, Federle TW, Stalmans M, Masscheleyn P (1995) Environmental Fate and Effects of Deedmac - a New Rapidly Biodegradable Cationic Surfactant for Use in Fabric Softeners. Chemosphere 30(6):1067-1083 doi:Doi 10.1016/0045-6535(95)00005-S

Gomez-Herrero E, Tobajas M, Polo A, Rodriguez JJ, Mohedano AF (2020) Toxicity and inhibition assessment of ionic liquids by activated sludge. Ecotoxicol Environ Saf 187:109836 doi:10.1016/j.ecoenv.2019.109836

Guerreiro P, Cupferman S, Lharidon J, Rozot R, Dalko-Csiba M (2023) Ethylated analogue of Zingerone: A new and eco-respectful preservative in cosmetics. Int J Cosmet Sci 45(2):187-197 doi:10.1111/ics.12830

Herrmann M, Menz J, Olsson O, Kummerer K (2015) Identification of phototransformation products of the antiepileptic drug gabapentin: Biodegradability and initial assessment of toxicity. Water Res 85:11-21 doi:10.1016/j.watres.2015.08.004
Jurado E, Fernandez-Serrano M, Nunez-Olea J, Luzon G, Lechuga M (2009) Acute toxicity and relationship between metabolites and ecotoxicity during the biodegradation process of non-ionic surfactants: fatty-alcohol ethoxylates, nonylphenol polyethoxylate and alkylpolyglucosides. Water Sci Technol 59(12):2351-8 doi:10.2166/wst.2009.266
Mena IF, Diaz E, Palomar J, Rodriguez JJ, Mohedano AF (2020) Cation and anion effect on the biodegradability and toxicity of imidazolium- and choline-based ionic liquids. Chemosphere 240:124947 doi:10.1016/j.chemosphere.2019.124947
Mengata Mengounou G, Moukengue Imano A, Sosso Mayi OT, Abomo Beyeme YY, Bekele Massako CF, Tchamdjio Nkouetcha E (2022) Biodegradability and ecotoxicity of bio-insulating oils in aqueous and soil environments in Douala, Cameroon. Scientific African 18 doi:https://doi.org/10.1016/j.sciaf.2022.e01413
Menz J, Baginska E, Arrhenius A, Haiss A, Backhaus T, Kummerer K (2017) Antimicrobial activity of pharmaceutical cocktails in sewage treatment plant effluent - An experimental and predictive approach to mixture risk assessment. Environ Pollut 231(Pt 2):1507-1517 doi:10.1016/j.envpol.2017.09.009
Murín M, Gavora J, Drastichová I, Dušková E, Madsen T, Torslov J, Damborg A, Tyle H, Pedersen F (1997) Aquatic hazard and risk assessment of two selected substances produced in high volumes in the Slovak Republic. Chemosphere 34(1):179-190 doi:Doi 10.1016/S0045-6535(96)00357-8
Mustahil NA, Baharuddin SH, Abdullah AA, Reddy AVB, Abdul Mutalib MI, Moniruzzaman M (2019) Synthesis, characterization, ecotoxicity and biodegradability evaluations of novel biocompatible surface active lauroyl sarcosinate ionic liquids. Chemosphere 229:349-357 doi:10.1016/j.chemosphere.2019.05.026
Pedrazzani R, Ceretti, E, Zerbini, I, Casale, R, Gozio, E, Bertanza, G, Gelatti, U, Donato, F, Feretti, D (2012) Biodegradability, toxicity and mutagenicity of detergents: Integrated experimental evaluations. . Ecotoxicol Environ Saf 84:274-281 doi:https://doi.org/10.1016/j.ecoenv.2012.07.023
Peric B, Sierra J, Marti E, Cruanas R, Garau MA, Arning J, Bottin-Weber U, Stolte S (2013) (Eco)toxicity and biodegradability of selected protic and aprotic ionic liquids. J Hazard Mater 261:99-105 doi:10.1016/j.jhazmat.2013.06.070
Pessala P, Keranen J, Schultz E, Nakari T, Karhu M, Ahkola H, Knuutinen J, Herve S, Paasivirta J, Ahtiainen J (2009) Evaluation of biodegradation of nonylphenol ethoxylate and lignin by combining toxicity assessment and chemical characterization. Chemosphere 75(11):1506-11 doi:10.1016/j.chemosphere.2009.02.011
Piętka-Ottlik M, Frąckowiak R, Maliszewska I, Kolwzan B, Wilk KA (2012) Ecotoxicity and biodegradability of antielectrostatic dicephalic cationic surfactants. Chemosphere 89(9):1103-11 doi:10.1016/j.chemosphere.2012.05.090
Puhlmann N, Olsson O, Kummerer K (2024) How data on transformation products can support the redesign of sulfonamides towards better biodegradability in the environment. Sci Total Environ 921:171027 doi:10.1016/j.scitotenv.2024.171027
Radošević K, Bubalo MC, Srcek VG, Grgas D, Dragicevic TL, Redovnikovic IR (2015) Evaluation of toxicity and biodegradability of choline chloride based deep eutectic solvents. Ecotoxicol Environ Saf 112:46-53 doi:10.1016/j.ecoenv.2014.09.034
Reynolds L, Blok J, de Morsier A, Gerike P, Wellens H, Bontinck WJ (1987) Evaluation of the toxicity of substances to be assessed for biodegradability. Chemosphere 16:2259-2277
Russo L, Rizzo L, Belgiorno V (2012) Ozone oxidation and aerobic biodegradation with spent mushroom compost for detoxification and benzo(a)pyrene removal from contaminated soil. Chemosphere 87(6):595-601 doi:10.1016/j.chemosphere.2012.01.012
Sági G, Bezsenyi A, Kovacs K, Klatyik S, Darvas B, Szekacs A, Mohacsi-Farkas C, Takacs E, Wojnarovits L (2018) Radiolysis of sulfonamide antibiotics in aqueous solution: Degradation efficiency and assessment of antibacterial activity, toxicity and biodegradability of products. Sci Total Environ 622-623:1009-1015 doi:10.1016/j.scitotenv.2017.12.065

Sanchis S, Polo AM, Tobajas M, Rodriguez JJ, Mohedano AF (2013) Degradation of chlorophenoxy herbicides by coupled Fenton and biological oxidation. Chemosphere 93(1):115-22 doi:10.1016/j.chemosphere.2013.04.097

Steger-Hartmann T, Lange R, Schweinfurth H (1999) Environmental risk assessment for the widely used iodinated X-ray contrast agent iopromide (Ultravist). Ecotoxicol Environ Saf 42(3):274-81 doi:10.1006/eesa.1998.1759

Stolte S, Steudte S, Areitioaurtena O, Pagano F, Thoming J, Stepnowski P, Igartua A (2012) Ionic liquids as lubricants or lubrication additives: an ecotoxicity and biodegradability assessment. Chemosphere 89(9):1135-41 doi:10.1016/j.chemosphere.2012.05.102

Straub JO (2009) An environmental risk assessment for oseltamivir (Tamiflu) for sewage works and surface waters under seasonal-influenza- and pandemic-use conditions. Ecotoxicol Environ Saf 72(6):1625-34 doi:10.1016/j.ecoenv.2008.09.011

Strotmann U, Butz B, Bias WR (1993) The dehydrogenase assay with resazurin: practical performance as a monitoring system and Ph-dependent toxicity of phenolic compounds. Ecotoxicol Environ Saf 25(1):79-89 doi:10.1006/eesa.1993.1009

Strotmann U, Eglsäer H, Pagga U (1994) Development and evaluation of a growth inhibition test with sewage bacteria for assessing bacterial toxicity of chemical compounds. Chemosphere 28(4):755-766 doi:10.1016/0045-6535(94)90229-1

Strotmann U, Pastor Flores D, Konrad O, Gendig C (2020) Bacterial Toxicity Testing: Modification and Evaluation of the Luminescent Bacteria Test and the Respiration Inhibition Test. Processes 8(11):1349 doi:https://doi.org/10.3390/pr8111349

Strotmann U, Weisbrodt W (1994) Wastewater treatment and integrated environmental protection at the BASF AG in Ludwigshafen, Germany. Water Sci Technol 29(8):185-192

Weltman R, Hargreaves T, Knight B, Wardrope L (2011) Assessment of the environmental fate and effects of the PPARgamma receptor agonist, pioglitazone. Chemosphere 83(4):391-9 doi:10.1016/j.chemosphere.2010.12.090

Wilde ML, Schneider M, Kummerer K (2017) Fenton process on single and mixture components of phenothiazine pharmaceuticals: Assessment of intermediaries, fate, and preliminary ecotoxicity. Sci Total Environ 583:36-52 doi:10.1016/j.scitotenv.2016.12.184
